# Rat models of postintracerebral hemorrhage pneumonia induced by nasal inoculation with *Klebsiella pneumoniae* or intratracheal inoculation with LPS

**DOI:** 10.3389/fimmu.2024.1477902

**Published:** 2025-01-08

**Authors:** Ruihua Wang, Changlian Gan, Rui Mao, Yang Chen, Ru Yan, Geng Li, Tianqin Xiong, Jianwen Guo

**Affiliations:** ^1^ Research Team of Prevention and Treatment of Cerebral Hemorrhage Applying Chinese Medicine, The Second Affiliated Hospital of Guangzhou University of Chinese Medicine, Guangzhou, China; ^2^ School of Traditional Dai Medicine, West Yunnan University of Applied Science, Xishuangbanna, China; ^3^ The Second Clinical College of Guangzhou University of Chinese Medicine, Guangzhou, China; ^4^ Department of Bioinformatics, State Key Laboratory of Dampness Syndrome of Chinese Medicine, The Second Affiliated Hospital of Guangzhou University of Chinese Medicine, Guangzhou, China; ^5^ State Key Laboratory of Quality Research in Chinese Medicine, Institute of Chinese Medical Sciences, University of Macau, Macao, Macao SAR, China; ^6^ Laboratory Animal Center, Guangzhou University of Chinese Medicine, Guangzhou, China; ^7^ School of Pharmaceutical Sciences, Guangzhou University of Chinese Medicine, Guangzhou, China; ^8^ State Key Laboratory of Traditional Chinese Medicine Syndrome, Department of Neurology, Guangdong Provincial Academy of Chinese Medical Sciences, Guangdong Provincial Hospital of Chinese Medicine, The Second Affiliated Hospital of Guangzhou University of Chinese Medicine, Guangzhou, China

**Keywords:** intracerebral hemorrhage, stroke-associated pneumonia, *Klebsiella pneumoniae*, lipopolysaccharide, 16S rRNA sequencing and untargeted metabolomics

## Abstract

**Background:**

A stable and reproducible experimental bacterial pneumonia model postintracerebral hemorrhage (ICH) is necessary to help investigating the pathogenesis and novel treatments of Stroke-associated pneumonia (SAP).

**Aim:**

To establish a Gram-negative bacterial pneumonia-complicating ICH rat model and an acute lung injury (ALI)-complicating ICH rat model.

**Methods:**

We established two standardized models of post-ICH pneumonia by nasal inoculation with *Klebsiella pneumoniae* (*Kp*) or intratracheal inoculation with lipopolysaccharide (LPS). Survival and neurological scores were monitored. Magnetic resonance imaging was performed to evaluate hematoma volume. Abdominal aortic blood was collected for leukocyte counting, serum was isolated to determine concentrations of S100β and proinflammatory cytokines using ELISAs. Histopathological changes of brain, lung and gut were assessed using hematoxylin−eosin staining. Lung was isolated for immunofluorescence staining for myeloperoxidase (MPO). Bronchoalveolar lavage fluid was collected for leukocyte counting, and supernatant was prepared to measure MPO activity. Ileum was isolated for immunofluorescence staining for tight junction proteins ZO-1 and γδ TCRs/IL-17A and for Alcian blue–nuclear fast red staining of acidic mucins. Feces were collected, 16S rRNA sequencing, untargeted metabolomics and Spearman’s correlation analyses were performed to explore changes of gut microbiota, metabolites and their interactions.

**Results:**

In *Kp*-induced bacterial pneumonia-complicating ICH rats, we demonstrated that *Kp* challenge caused more severe neurological deficits, brain damage, neuroinflammation, and aggravated pneumonia and lung injury. Disruptions of the intestinal structure and gut barrier and the reductions of the protective intestinal IL-17A-producing γδT cells were also observed. *Kp* challenge exacerbated the gut microbiota dysbiosis and fecal metabolic profile disorders, which were characterized by abnormal sphingolipid metabolism especially elevated ceramide levels; increased levels of neurotoxic quinolinic acid and an upregulation of tryptophan (Trp)–serotonin–melatonin pathway. Spearman’s correlation analyses further revealed that the reduction or depletion of some beneficial bacteria, such as *Allobaculum* and *Faecalitalea*, and the blooming of some opportunistic pathogens, such as *Turicibacter*, *Dietzia*, *Corynebacterium* and *Clostridium_sensu_stricto_1* in *Kp*-induced SAP rats were associated with the disordered sphingolipid and Trp metabolism. Using an LPS-induced ALI complicating ICH model, we also characterized SAP-induced brain, lung and gut histopathology injuries; peripheral immune disorders and intense pulmonary inflammatory responses.

**Conclusions:**

These two models may be highly useful for investigating the pathogenesis and screening and optimizing potential treatments for SAP. Moreover, the differential genera and sphingolipid or Trp metabolites identified above seem to be promising therapeutic targets.

## Introduction

Stroke is the second leading cause of death and the third leading cause of death and disability worldwide (1). Intracerebral hemorrhage (ICH) accounts for 9–27% of stroke cases (2) and is the most lethal and least treatable subtype (3). Stroke-associated pneumonia (SAP) is one of the major complications after stroke and is associated with significantly increased health care costs, poor functional outcomes and mortality (4). The incidence of SAP in ICH patients continues to be high, ranging from 14.3–31.1% (5, 6). Currently, the clinical strategies used to prevent or treat SAP are based on the use of antibiotics. Major clinical trials have shown that prophylactic antibiotics have no beneficial effects on functional outcomes or mortality (7). Despite being effective for the treatment of infections, they need to be used cautiously because of potentially harmful adverse effects and an alarming increase in antibiotic-resistant strains of bacteria (8). Thus, more effective therapeutic strategies that target the underlying pathogenesis of SAP are urgently needed.

Animal models are necessary to help reveal the pathogenesis and validate new interventions that might alleviate SAP. Various experimental models of SAP have been developed in practice to mimic human clinical scenarios. Mice can develop spontaneous bacterial infections and pneumonia due to stroke-induced immunodeficiency, as well as the translocation of the gut microbiota to the lungs (9–11). However, some studies have reported conflicting results (12, 13). Moreover, the incidence and severity of spontaneous pneumonia vary depending on genetic background (14), environmental factors such as animal housing facility conditions that may affect the microbiota and thus systemic immune responses (15–17), and the choice of stroke model (18). A model inducing standardized bacterial pneumonia is desirable to increase the reproducibility and predictability of SAP studies. Currently, intranasally inoculated *Streptococcus pneumoniae* (*Sp*) or *Klebsiella pneumoniae* (*Kp*) (19, 20) or intratracheally injected *Sp*-induced bacterial pneumonia complicating ischemic stroke models (21) have been successfully established. However, a stable and reproducible experimental bacterial pneumonia model post-ICH is not currently available.

A systematic review of 15 studies involving 7968 stroke patients suggested that aerobic Gram-negative bacilli (38%) and Gram-positive cocci (16%) were most frequently isolated from sputum, tracheal aspirate and blood cultures, with *Enterobacteriaceae* (*Kp*, *Escherichia coli*), *Staphylococcus aureus*, *Pseudomonas aeruginosa*, *Acinetobacter baumannii* and *Sp* identified as the most common organisms responsible for pneumonia complicating stroke (22).

Considering that *Kp* is the pathogen most frequently isolated from SAP, in this study, we established a Gram-negative bacterial pneumonia complicating ICH model via nasal inoculation with *Kp* after collagenase-induced ICH in rats (23). Since *Kp* is a biosafety level 2 (BSL-2) opportunistic pathogen associated with infections (24), the widespread use of a *Kp*-induced bacterial pneumonia complicating ICH model is limited because of biosafety concerns and the limited availability of high-level biosafety labs. Lipopolysaccharide (LPS), a key component of Gram-negative bacterial cell walls, has been extensively used to simulate Gram-negative bacterium-induced acute lung inflammation and lung injury (ALI) (25). Therefore, we further established LPS-induced ALI complicating ICH model via intratracheal inoculation with LPS after ICH. Using these two models, we characterized SAP−induced brain, lung, and gut histopathological injuries and peripheral and pulmonary immune disorders. We further performed 16S ribosomal RNA (rRNA) sequencing, untargeted metabolomics and Spearman’s correlation analyses to identify SAP-related gut microbial species, metabolites and functional pathways.

## Materials and methods

### Animals

For Experiments 1, 3, and 4, male Sprague−Dawley (SD) rats (200–240 g, 8–9 weeks old) of specific pathogen-free (SPF) grade were obtained from Guangdong Medical Laboratory Animal Center (Guangdong, China) and housed in the Laboratory Animal Center of Guangdong Provincial Hospital of Chinese Medicine under standard care conditions (temperature, 24 ± 2°C; relative humidity, 60 ± 5%; normal day/night cycle, 12/12 h; with food and water available ad libitum). For Experiment 2, male SD rats (200–240 g, 8–9 weeks old) of SPF grade were obtained from ZhuHai Bestest Biotechnology Co., Ltd. (Guangdong, China) and housed in the Laboratory Animal Center of Guangzhou University of Chinese Medicine under standard care conditions. All animal experiments were performed according to the Guidelines for the Care and Use of Laboratory Animals of the National Institutes of Health and protocols approved by the Institutional Ethics Committee of Guangzhou University of Chinese Medicine or Guangdong Provincial Hospital of Chinese Medicine.

### Study design

#### Experiment 1

ICH models were induced by injecting different doses of type VII collagenase (Sigma−Aldrich, USA) into the right caudate nucleus to investigate whether SD rats can develop secondary pulmonary infection and pneumonia after ICH. Twelve rats were randomly assigned to the following four groups (n=3 rats per group): the sham and 0.175 U, 0.35 U and 0.7 U type VII collagenase-induced ICH. At 7 days after ICH, brain and lung samples were collected to assess histopathological changes using hematoxylin−eosin (H&E) staining.

#### Experiment 2

Thirty-three rats were randomly assigned to the following three groups: the sham (n=10), ICH (n=9) and SAP (ICH *+ Kp*, n=14). Survival was monitored daily. The neurological score was recorded from Day 3 to Day 7 after ICH induction. At 7 days after ICH (4 days after *Kp* inoculation), serum was collected from the abdominal aortic blood to determine the concentration of S100β using ELISA. Histopathological changes in the brain, lung, ileum and colon were assessed via H&E staining. Lung tissues were isolated for immunofluorescence staining for myeloperoxidase (MPO). Bronchoalveolar lavage fluid (BALF) supernatant was prepared for measurement of MPO activity. Ileum sample was isolated for immunofluorescence staining for the tight junction proteins zonula occluden-1(ZO-1) and γδ T cell receptors (TCRs)/interleukin (IL)-17A and for Alcian blue–nuclear fast red (AB-NFR) staining of acidic mucins. Fecal sample was collected before sacrifice, 16S rRNA sequencing, untargeted metabolomics and Spearman’s correlation analyses were performed to explore the changes in the gut microbiota, metabolites and their interactions.

#### Experiment 3

Forty-five rats were randomly assigned to the following three groups (n=15 rats per group): the sham, 2 mg/kg LPS-SAP (ICH *+* 2 mg/kg LPS) and 3 mg/kg LPS-SAP (ICH *+* 3 mg/kg LPS) groups. At 4 days after ICH (1 day after LPS inoculation), serum was collected from the abdominal aortic blood to determine the concentrations of tumor necrosis factor-α (TNF-α), IL-1β and IL-6 using ELISAs. BALF was prepared for cell counting.

#### Experiment 4

Thirty rats were randomly assigned to the following two groups (n=15 rats per group): the sham and SAP (ICH *+* 3 mg/kg LPS). Survival, body weight and neurological scores were monitored daily. At 4 days after ICH (1 day after LPS inoculation), magnetic resonance imaging (MRI) was performed to detect the locations, boundaries and extent of the hematomas using susceptibility-weighted imaging (SWI) pulse sequences. Abdominal aortic blood was collected for leukocyte counting. Histopathological changes in the brain, lung and ileum were assessed via H&E staining.

### Induction of experimental ICH

Our experimental ICH procedure is based on a previously described method reported by Rosenberg et al. (23) with some modifications. Briefly, after being anesthetized with pentobarbital sodium (40 mg/kg, 1%) via intraperitoneal injection, SD rats were fixed in a stereotactic apparatus (RWD, Life Science, China) in the prone position. A total of 0.5 U type VII collagenase (Sigma−Aldrich, USA) prepared in 2 μL of TESCA buffer (Solarbio, China) containing 2.5 U/μL heparin sodium (Sigma−Aldrich, USA) was stereotactically injected into the right basal ganglia within 5 minutes (coordinates, 3.0 mm right lateral to the bregma, 6 mm ventral relative to the skull surface) using a 10 μL microsyringe needle (Gaoge Industry and Trade Co., Ltd., Shanghai, China). The needle was left in place for 5 minutes (min) and then slowly withdrawn. The sham rats underwent the same surgical procedures without the injection of collagenase, instead, 2 μL of TESCA buffer containing 2.5 U/μL heparin sodium was injected. Zea-Longa scoring was performed to assess brain damage the next day. Rats with Zea-Longa scores ranging from 1–3 were selected for nasal inoculation with *Kp* or intratracheal inoculation with LPS at 3 days after ICH.

### Bacterial culture and nasal inoculation with *Kp*

*K. pneumoniae* K6 (American Type Culture Collection #700603) was grown from frozen stocks on Mueller−Hinton (MH) agar plates (Solarbio, China) overnight at 37°C. Colonies were inoculated into MH broth medium (Solarbio, China) and cultured with shaking overnight. Cultures were diluted in MH broth until they reached the mid-logarithmic growth phase (OD600 nm, 0.7). Bacteria were pelleted via centrifugation, washed twice with 0.9% saline and diluted to a final concentration of 2×10^6^ colony-forming units (CFUs)/ml. 50 μL of the bacterial suspension was slowly injected into each nostril of each SAP rat after anesthetizing them with pentobarbital sodium, and 50 μL of the 0.9% saline alone (control) was slowly injected into each nostril of each sham or ICH rat. After inoculation, the rats were maintained upright and rotated for 2 min so that the *Kp* was uniformly distributed into the lungs. The rats were placed under an infrared heating lamp until they recovered from anesthesia. The inoculated animals were housed separately from the 0.9% saline-only controls.

### Intratracheal inoculation with LPS

The rats were anesthetized and placed in a supine position on a board. LPS (055:B5, Sigma, USA) was dissolved in 0.9% saline. 200 μL of LPS solution was injected into the trachea at a dosage of 2 mg/kg or 3 mg/kg using a 1 ml insulin syringe with a 29-gauge needle (BD, USA). After inoculation, the rats were kept upright and rotated for 2 min to ensure that the LPS was uniformly distributed into the lungs. The rats were placed under an infrared heating lamp until they recovered from anesthesia. The inoculated animals were housed separately from the 0.9% saline-only controls.

### Neurological function evaluation

The modified neurological severity score (mNSS) was used to evaluate the neurological deficits of the ICH rats. mNSS consists of 4 subtests that assess motor, sensory, balance and reflex functions. Neurological function was graded on a scale of 0–18. A higher score indicates worse neurological function (1–6 indicates mild injury, 7–12 indicates moderate injury, and 13–18 indicates severe injury) (26). All tests were conducted by an investigator who was blinded to the experimental groups.

### MRI examination and hematoma volume calculation

The MRI scans were performed using a 3.0-T MRI scanner system (MAGNETOM Prisma, Siemens Healthcare, Germany). After being deeply anesthetized, the rats were positioned prone with the head inside an 8-channel mouse coil (Chen Guang Medical Technologies Co., Ltd., Shanghai, China). SWI was acquired 4 days after ICH induction. The parameters are as follows: matrix, 192×192; FOV, 60 mm × 60 mm; TR, 30.0 ms; TE, 20.0 ms; flip angle, 15°; slice thickness, 1.0 mm; and slice gap, 0.2 mm. The hematoma volume was calculated according to the Coniglobus formula, where V = a × b × c × 1/2 at the largest hematoma level (a indicates the longest diameter of hematoma, b indicates the longest diameter perpendicular to a, and c indicates the number of layers with bleeding × slice thickness). All examinations and hematoma volume calculations were conducted by a qualified physician who was blinded to the experimental groups.

### Histopathological changes in the brain, lung and gut tissues

Histopathological changes in the brain, lung and gut tissues were assessed via H&E staining. The rats were deeply anesthetized and transcardially perfused with 200 mL of precooled 0.9% saline. The brain, lung, ileum and colon samples were isolated, fixed with 10% phosphate-buffered formalin, embedded in paraffin and sliced into 4−μm thick sections. After deparaffinization, the tissue sections were stained with HE according to the manufacturer’s instructions (Pinofei Biotechnology Co., Ltd., Wuhan, China). The sections were scanned with the PANNORAMIC MIDI II and viewed using SlideViewer (3DHISTECH, Hungary).

### Immunofluorescence staining

As previously described, the paraffin-embedded tissues were sliced into 4−μm thick sections. After deparaffinization and rehydration, the sections were immersed in 10 mM sodium citrate buffer (pH 6.0) (AR0024, Boster, Wuhan, China) for antigen retrieval by boiling in a microwave for 8 min, followed by an incubation in PBS supplemented with 0.3% Triton X-100 for 10 min and 10% goat serum (Sangon Biotech Co., Ltd., Shanghai, China) for 1 h at room temperature. For analysis of the expression of the tight junction protein ZO-1, ileum sections were subjected to an overnight incubation with a ZO-1 rabbit polyclonal antibody (1:300, Affinity, Cat# AF5145) at 4°C in a humidified environment. For the analysis of the expression of MPO, lung sections were subjected to an overnight incubation with aMPO rabbit monoclonal antibody (1:100, Abcam, Cat# ab208670) at 4°C in a humidified environment. The sections were subsequently washed with PBST and incubated with Alexa Fluor^®^ 555-conjugated goat anti-rabbit IgG (H+L), F(ab’)2 fragment (1:2000, Cell Signaling Technology, Cat# 4413S) at 37°C for 1 h. The ileum sections were incubated with the following antibodies at room temperature for 2 h in a humidified environment to analyze the number of γδ T cells and IL-17A expression: PE-conjugated mouse anti-rat γδ T-cell receptor (1:15, BD Pharmingen™, Cat#551802) and PE-Cyanine7-conjugated anti-rat IL-17A monoclonal antibody (1:15, eBioscience™, Cat# 25-7177-82). Finally, the sections were stained with DAPI and mounted with antifade medium (Cat# S2110; Solarbio, Beijing, China). Representative images were captured with a fluorescence microscope (Eclipse TS100, Nikon, Japan). The images were semiquantitated using ImageJ version 1.51 (NIH, USA).

### AB-NFR staining

AB-NFR staining was performed to analyze the expression of acidic mucins according to the manufacturer’s instructions (Cat #C0155S, Beyotime Biotechnology, Haimen, China). Briefly, the paraffin-embedded ileum samples were sliced into 4−μm thick sections. After deparaffinization, the sections were rehydrated through alcohol gradient washes, stained with an Alcian blue solution (pH 2.5) for 1 h in a humidified environment, rinsed with distilled water and stained with nuclear fast red solution (pH 2.5) for 5 min. The sections were then rinsed with distilled water again and gradually dehydrated by gradient alcohol. Finally, the sections were cleared in xylene and observed under an inverted microscope (Eclipse TS100, Nikon, Japan) after being sealed with neutral gum (Solarbio, Beijing, China). The relative density of positive area in AB-PAS staining was calculated using ImageJ version 1.51 (NIH, USA).

### Routine blood testing

2 mL of abdominal aortic blood was collected from the rats in Experiment 4 into a 5 mL EDTA-K2 anticoagulant tube (REF# 683050202, Improve Medical, Guangzhou, China) at 4 days after ICH. The white blood cell count was analyzed using an automatic animal blood cell analyzer (BC-2800 Vet, Mindray Animal Medical, Shenzhen, China).

### Enzyme-linked immunosorbent assay

5 mL of abdominal aortic blood was collected into 5 mL Gel & Clot Activator Tubes (REF# 623050202, Improve Medical, Guangzhou, China) at 7 days after ICH from the rats in Experiment 2 or at 4 days after ICH from the rats in Experiment 3. The concentrations of S100β, TNF-α, IL-1β and IL-6 in the serum were determined using a S100β SimpleStep ELISA^®^ Kit (ab234573, Abcam, Cambridge, United Kingdom), a rat TNF-α ELISA Kit (EK0526, BOSTER, Wuhan, China), a rat IL-1β ELISA Kit (SEKR-0002, Solarbio, Beijing, China) and a rat IL-6 ELISA Kit (CSB-E04640r, CUSABIO, Wuhan, China), respectively, according to the manufacturers’ instructions. The absorbance value at 450 nm was measured using a microplate reader (Multiskan FC, Thermo Fisher Scientific, USA).

### Measurement of leukocyte counts and MPO activity in BALF

After right main bronchus ligation, BALF was collected from the left lungs of the rats in Experiment 2 at 7 days after ICH or of the rats in Experiment 3 at 4 days after ICH using three consecutive instillations of 1 mL of precooled 0.9% saline. Total and differential leukocyte counts in the BALF collected from the rats in Experiment 3 were analyzed using an automatic animal blood cell analyzer (BC-2800 Vet, Mindray Animal Medical, Shenzhen, China). The BALF from the rats in Experiment 2 was stored at −80°C. After centrifugation at 1,500 × g for 5 min at 4°C, MPO activity in the undiluted cell-free BALF was assessed using a MPO activity assay kit (BB-47262, BestBio, Shanghai, China) according to the manufacturer’s instructions. The absorbance value at 460 nm was measured using a microplate reader (Multiskan FC, Thermo Fisher Scientific, USA). The results are presented as MPO units per liter of BALF (U/L).

### DNA extraction and 16S rRNA sequencing

Total genomic DNA was extracted from the fecal samples using the E.Z.N.A.^®^ soil DNA kit (Omega Biotek, USA). The quality of the samples was monitored on 1% agarose gels, and the purity and concentration were measured using a NanoDrop 2000 UV–Vis spectrophotometer (Thermo Scientific, USA). The DNA was diluted to 1 ng/µl, and the V3–V4 regions of the bacterial 16S rRNA gene were amplified using the barcoded primers 338 F (5’-ACTCCTACGGGAGGCAGCAG-3’) and 806 R (5’-GGACTACHVGGGTWTCTAAT-3’) with an ABI GeneAmp^®^ 9700 PCR thermocycler (ABI, CA, USA). After extraction from the 2% agarose gel, the PCR products were purified using an AxyPrep DNA gel extraction kit (Axygen Biosciences, USA) and quantified using a QuantiFluor™-ST fluorometer (Promega, USA). The amplicons were mixed in equimolar concentrations and used for library construction with the TruSeq™ DNA Sample Prep Kit (Illumina, USA) according to the manufacturer’s instructions. Paired-end sequencing was then performed on the Illumina MiSeq PE300 platform/NovaSeq PE250 platform (Illumina, USA) according to the standard protocol of Majorbio Bio-Pharm Technology Co. Ltd. (Shanghai, China). The raw sequencing data were screened, quality-filtered using fastp version 0.19.6 (https://github.com/OpenGene/fastp) and assembled using FLASH version 1.2.11 (https://ccb.jhu.edu/software/FLASH/index.shtml). Operational taxonomic units (OTUs) with 97% similarity were clustered in UPARSE version 11 (http://www.drive5.com/uparse/), with chimeric sequences excluded. The representative sequences were classified into organisms via a naïve Bayesian model using the Ribosomal database project classifier version 2.13 (https://sourceforge.net/projects/rdp-classifier/) against the 16S rRNA database Silva version 138 (https://www.arb-silva.de/). The analysis of the significant differences in the gut microbial community composition among the groups was further performed using the free online tools of the Majorbio cloud platform (www.majorbio.com, Majorbio Biopharm Technology Co., Ltd.).

### Metabolomic analysis of fecal samples

An untargeted metabolomics approach was employed. Briefly, 50 mg fecal sample was added to a 2 mL centrifuge tube containing 400 μL of extraction solution (methanol/water, 4:1, v/v), 0.02 mg/mL the L-2-chlorophenylalanine internal standard and a 6 mm diameter grinding bead. The samples were ground for 6 min at -10°C and 50 Hz using a Wonbio-96c frozen tissue grinder (Shanghai Wanbo Biotechnology Co., Ltd., China), followed by low-temperature ultrasonic extraction for 30 min (5°C, 40 kHz). The samples were incubated at -20°C for 30 min and centrifuged for 15 min (4°C, 13000 × g), after which the supernatant was transferred to an injection vial for liquid chromatography (LC)–mass spectrometry (MS)/MS analysis using a Thermo UHPLC-Q Exactive HF-X system according to the standard protocol of Majorbio Bio-Pharm Technology Co. Ltd. (Shanghai, China). A pooled quality control sample (QC) was prepared by mixing equal volumes of all samples to monitor the stability of the analysis. Chromatography was performed on an ACQUITY HSS T3 column (Waters, USA) with mobile phases consisting of 0.1% formic acid in water/acetonitrile (95:5, v/v, solvent A) and 0.1% formic acid in acetonitrile/isopropanol/water (47.5:47.5:5, v/v, solvent B). The flow rate was 0.40 mL/min, and the column temperature was 40°C. The MS analysis was conducted using a Thermo UHPLC-Q Exactive HF-X mass spectrometer equipped with an electrospray ionization (ESI) source operating in positive and negative modes. The optimal conditions used were as follows: source temperature of 425°C; ion-spray voltage floating (ISVF) of -3500 V or 3500 V in negative or positive mode, respectively; sheath gas flow rate of 50 arb; aux gas flow rate of 13 arb; normalized collision energy of 20, 40, and 60V rolling for MS/MS. The full MS resolution was 60000, and the MS/MS resolution was 7500. Data acquisition was performed in data-dependent acquisition (DDA) mode. The mass scan range was 70–1050 m/z. The raw files obtained were imported into Progensis QI (Waters Corporation, USA) software for data processing. The metabolites were identified by searching the HMDB (http://www.hmdb.ca/), Metlin (https://metlin.scripps.edu/) and Majorbio databases. The data matrix obtained was uploaded to the Majorbio cloud platform (https://majorbio.com) for data analysis. First, the data matrix was preprocessed as follows: At least 80% of the metabolic features detected in any set of samples were retained. After filtering, for specific samples whose metabolite levels were below the lower limit of quantification, the minimum metabolite value was estimated, and each metabolic signature was normalized to the sum. The response intensities of the sample MS peaks were normalized using the sum normalization method to obtain the normalized data matrix and to reduce the errors caused by sample preparation and instrument instability. The variables of the QC samples with a relative standard deviation (RSD) > 30% were excluded, and log10 transformation was performed to obtain the final data matrix for subsequent analysis. The R package ropls version 1.6.2 was subsequently used to perform principal component analysis (PCA) and orthogonal least partial squares discriminant analysis (OPLS-DA), with 7-cycle interactive validation conducted to evaluate the stability of the model. Metabolites with VIP>1 and p<0.05 were determined to be significantly different metabolites based on the variable importance in the projection (VIP) values obtained from the OPLS-DA model and the p values generated by Student’s t test. Differentially abundant metabolites were mapped to their biochemical pathways through pathway topology analyses using the Kyoto Encyclopedia of Genes and Genomes (KEGG) database (http://www.genome.jp/keg/) to obtain additional insights into the underlying biological mechanisms associated with SAP. The identified differential pathways between two groups are presented according to pathway impact values from the pathway topology analysis, with the most impacted pathways marked in red, while the P value represents the enrichment of certain metabolites in a pathway (P<0.05 is indicative of significant enrichment). The Python package scipy version 1.0.0 (https://docs.scipy.org/doc/scipy/) was used to perform enrichment analyses to obtain the most relevant biological pathways of SAP. Spearman’s correlation analyses were performed to explore the associations between differentially abundant genera and metabolites.

### Statistical analysis

All omics data were analyzed using the free online tools of the Majorbio cloud platform (www.majorbio.com, Majorbio Biopharm Technology Co., Ltd.). For the analysis of nonomics data, GraphPad software version 9.5.1 (GraphPad, San Diego, CA, USA) was used. The data are presented as the means ± SEMs. The statistical significance of differences between two groups was determined using two-tailed unpaired Student’s t tests. For comparisons of three or more groups, when the data both satisfied a normal distribution and homogeneity of variance, one-way analysis of variance (ANOVA) followed by Tukey’s or Bonferroni’s multiple comparison test were conducted. When the data were normally distributed but not homogeneous, the Brown–Forsythe test and Welch ANOVA test followed by Dunnett’s T3 multiple comparison test were used. When the data did not satisfy a normal distribution and homogeneity of variance, the Kruskal−Wallis H test followed by Dunnett’s T3 multiple comparison test were conducted. Two-way ANOVA followed by Bonferroni’s multiple comparisons test were used to evaluate the entire time course variation. For the survival rate analysis, Kaplan–Meier analysis with the log-rank test was used. P < 0.05 was considered to indicate statistical significance.

## Results

### Not all SD rats develop spontaneous pneumonia after ICH, and the severity of spontaneous pneumonia varies even within a group

We established collagenase-induced ICH models by injecting different doses of type VII collagenase into the right caudate nucleus according to the protocol of Rosenberg et al. (23) to investigate whether SD rats can spontaneously develop pneumonia after ICH. Brain and lung samples were collected 7 days after ICH induction, and histopathological changes were assessed via H&E staining (**Supplementary Figures 1A, B**). As expected, the extent of the brain lesions differed substantially among the 3 models and depended on the amount of enzyme injected, since the injection of 0.7 U collagenase caused a larger focal area of damage, whereas the injections of 0.35 U and 0.175 U collagenase induced much smaller or even no apparent damage (**Supplementary Figure 1A**). Lung histological changes were also evaluated. As shown in **Supplementary Figure 1B**, significant lung histological deterioration, such as alveolar wall thickening, edema and hemorrhage, was observed in some but not all of the rats in the 3 aforementioned models. Furthermore, the severity of spontaneous pneumonia varies even within a group. Hence, a stable and reproducible bacterial pneumonia model post-ICH is desirable to investigate the pathogenesis of and novel treatments for SAP.

### Establishment of a model of Gram-negative bacterial pneumonia complicating ICH via nasal inoculation with *Kp* after ICH

**Figure 1:**
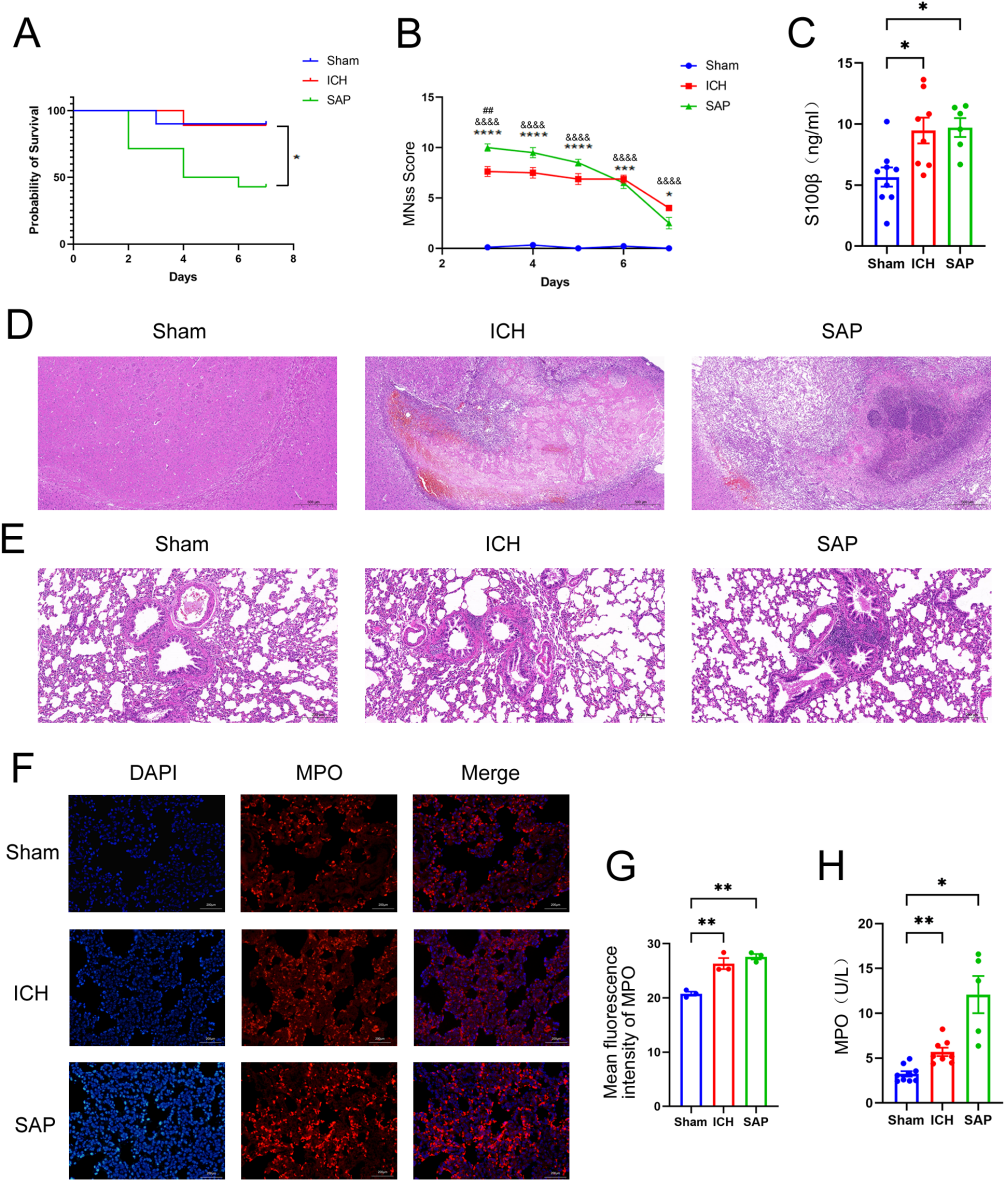
Changes in survival rates, mNSS, the serum S100β concentration, the histopathology of brain and lung tissues, and the expression and activity of MPO in the lungs in *Kp*-induced Gram-negative bacterial pneumonia-complicating ICH (SAP) rats. **(A)** Survival rates of sham-operated control, ICH and SAP rats. n = 9–14 rats per group. **(B)** Changes in the mNSSs of sham-operated control, ICH and SAP rats. Data were recorded from Day 3 to Day 7 after ICH. n = 6–9 rats per group. **(C)** ELISA of S100β levels in the serum of sham-operated control, ICH and SAP rats 7 days after ICH induction. n = 6–8 rats per group. **(D, E)** Representative images of H&E-stained brain **(D)** and lung **(E)** sections from sham-operated control, ICH and SAP rats at 7 days after ICH. n = 6–7 rats per group. Scale bars, 500 μm **(D)** and 200 μm **(E, F)** Immunofluorescence staining showing MPO-positive cells in the lungs of sham-operated control, ICH and SAP rats at 7 days after ICH. n = 3 rats per group. Scale bars, 200 μm. **(G)** The relative mean fluorescence intensity of MPO. **(H)** MPO activity in the BALF of sham-operated control, ICH and SAP rats at 7 days after ICH. n = 5–9 rats per group. The data are presented as the means ± SEMs. For **(A)**, Kaplan–Meier analysis with the log-rank test was used. *P<0.05 for SAP vs. ICH. For **(B)**, two-way ANOVA with Bonferroni’s multiple comparisons test was used. *P<0.05, ***P<0.001, and ****P<0.0001 SAP vs. sham-operated control; ^&&&&^P<0.0001 ICH vs. sham-operated control; ^##^P<0.01 SAP vs. ICH. For **(C)**, one-way ANOVA with Bonferroni’s multiple comparisons test was used, *P<0.05. For **(G)**, one-way ANOVA with Turkey’s multiple comparisons test was used, **P<0.01. For **(H)**, the Brown–Forsythe test and Welch’s ANOVA followed by Dunnett’s T3 multiple comparison test was used. *P < 0.05 and **P < 0.01. Sham, sham-operated control; ICH, collagenase-induced intracerebral hemorrhage model; SAP, *Kp*-induced Gram-negative bacterial pneumonia complicating ICH model.

We established a rat Gram-negative bacterial pneumonia complicating ICH model via nasal inoculation with 2×10^5^ CFUs of *Kp* 3 days after ICH. The survival rate was 42.9% 7 days after ICH induction (4 days post-*Kp* inoculation, **Figure 1A**). mNSS assessments were performed from Day 3 (the day of *Kp* inoculation) to Day 7 after ICH to evaluate ICH-induced neurological impairment. Compared with the sham-operated controls, both the ICH and SAP groups presented higher mNSSs. Moreover, compared with the ICH group, the SAP group presented higher mNSSs at Day 3 after ICH, which indicates more severe neurological deficits (**Figure 1B**). Serum S100β is produced mainly by leakage from activated and disrupted astrocytes in the central nervous system (CNS) into the peripheral blood stream in the case of blood–brain barrier (BBB) disruption. Elevated peripheral S100β levels have been detected in both ICH patients (27) and experimental animals (28) and are often considered a biomarker of brain damage after ICH. We found that the serum S100β level was significantly elevated in both the ICH and SAP groups compared with the sham-operated control group, while a significant difference was not observed between the ICH and SAP groups (**Figure 1C**), indicating that SAP indeed induced brain damage and BBB disruption at Day 7 after ICH. Histological changes in the brain were also evaluated. As shown in **Figure 1D**, ICH induced significant histological deterioration, and the lesions included a large mosaic-like central zone with pale staining, where extravasated erythrocytes were separated by fragments of necrotic parenchyma, and a perihematomal area, which was a narrow band of poorly stained parenchyma containing either viable or necrotic glial and neuronal cells, with intense infiltration of inflammatory cells. Compared with the ICH group, the SAP group exhibited more severe histological deterioration characterized by more intense infiltration of inflammatory cells not only in the perihematomal area but also in the central zone. Taken together, these data reveal that SAP can induce more serious neurological deficits, brain damage and neuroinflammation in lesions.

### *Kp* challenge exacerbates pulmonary inflammation and lung injury in ICH rats

We evaluated histopathological changes to investigate whether *Kp* challenge could induce pneumonia and lung injury in ICH rats (**Figure 1E**). Lung sections from the sham-operated control group were morphologically normal, whereas those from the ICH group presented focal mild peribronchiolar edema and inflammation. However, SAP rats developed more severe peribronchiolar edema and intense infiltration of inflammatory cells. The rapid influx of neutrophils into the interstitium and bronchoalveolar spaces and subsequent overactivation play key roles in the development and progression of ALI (29, 30). MPO, a member of the heme peroxidase enzyme family, is predominantly secreted by activated neutrophils (31), the MPO activity can be used as a marker of neutrophil activation (32). Thus, we performed immunofluorescence staining to analyze the expression of MPO in the lungs of the three groups. As shown in **Figures 1F, G**, both ICH and SAP groups displayed significantly higher levels of MPO than that in the sham-operated control group. The MPO activity in the BALF was also examined. The result revealed that the MPO activity in the BALF of the ICH and SAP groups was significantly higher than that in the sham-operated control group. Moreover, although a significant difference was not observed in pairwise comparisons, the SAP group displayed higher MPO activity than that in the ICH group (**Figure 1H**). These data indicate that *Kp* challenge aggravates pneumonia and lung injury in ICH rats.

### Disruptions of the intestinal structure and gut barrier and the reductions of the protective intestinal IL-17A-producing γδT cells were observed in *Kp*-induced SAP rats

**Figure 2:**
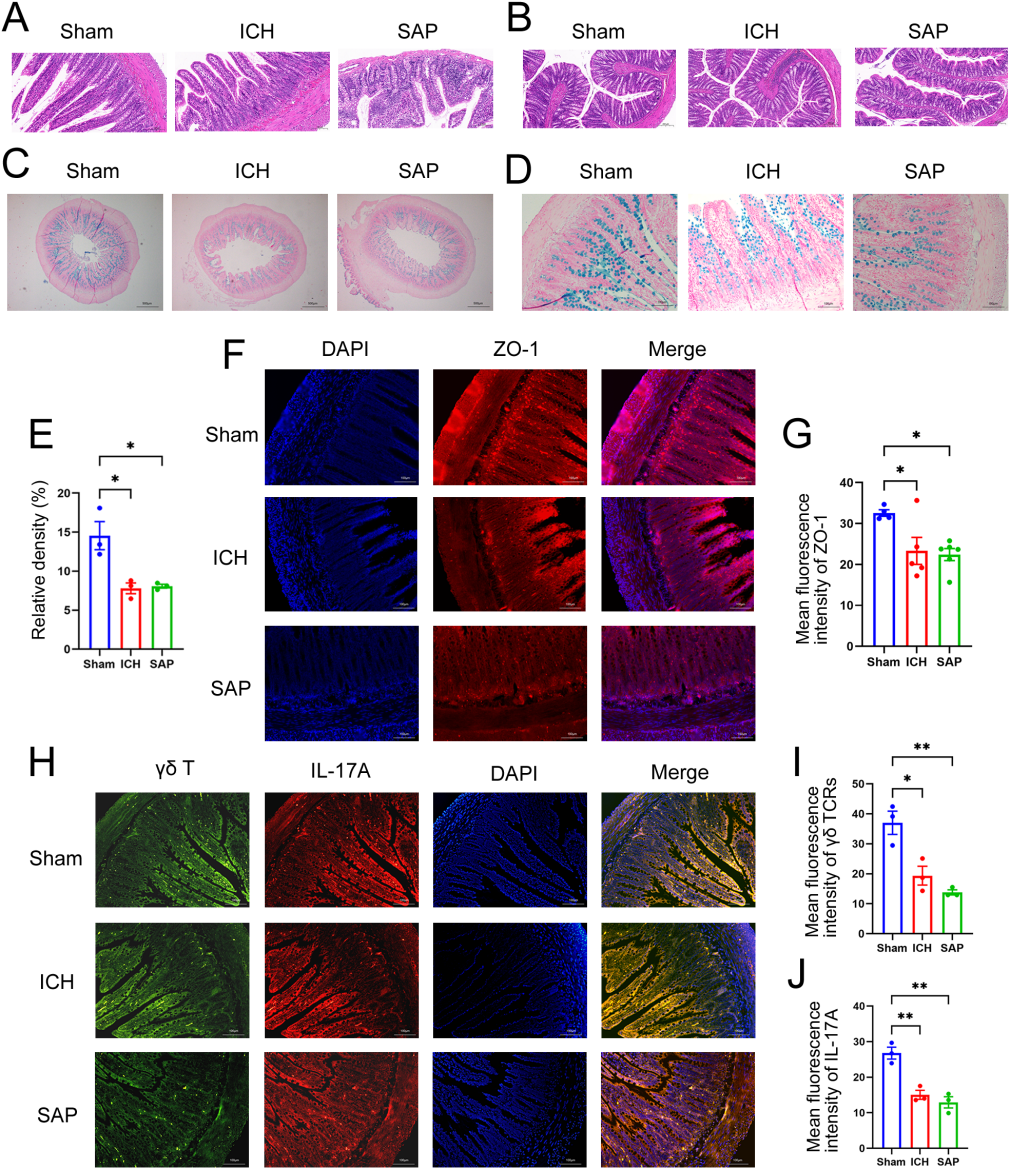
*Kp*-induced SAP rats exhibited the intestinal structure and gut barrier damages and the reductions of the protective intestinal IL-17A-producing γδT cells at 7 days after ICH induction. **(A, B)** Representative images of H&E staining of ileum **(A)** and colon sections **(B)** from sham-operated control, ICH and SAP rats. n = 4–7 rats per group. Scale bars, 100 μm **(A)** and 200 μm **(B–D)** AB–NFR staining of acidic mucins in the ileums of sham-operated control, ICH and SAP rats. n = 3 rats per group. Scale bars, 500 μm **(C)** and 100 μm **(D, E)** Proportion of positive area of AB-PAS staining of ileum tissues. **(F)** Immunofluorescence staining for ZO-1 in the ileums of sham-operated control, ICH and SAP rats. n = 3–5 rats per group. Scale bars, 100 μm. **(G)** The relative mean fluorescence intensity of ZO-1. **(H)** Immunofluorescence staining showing γδ TCR- and IL-17A-positive cells in the ileums of sham-operated control, ICH and SAP rats. n = 3 rats per group. Scale bars, 100 μm. **(I, J)** The relative mean fluorescence intensity of γδ TCR **(I)** or IL-17A **(J)**. The data are presented as the means ± SEMs. For **(E, I, J)**, one-way ANOVA and Turkey’s multiple comparisons test were used. For **(G)**, one-way ANOVA and Bonferroni’s multiple comparisons test were used. *P < 0.05, **P < 0.01.

A growing body of evidence suggests that both ischemic stroke and hemorrhagic stroke can rapidly induce gastrointestinal dysfunction, intestinal structure and gut barrier destructions (11, 33). The intestinal histopathological changes were also evaluated. As shown in **Figures 2A, B**, ICH alone did not induce the significant histological deterioration of ileum or colon tissues, probably due to a self-recovery mechanism at 7 days after ICH. However, SAP rats indeed developed obvious histological changes characterized by a thinner layer of epithelium and muscularis mucosae, thickened and shortened villi, a loss of crypts and glands, edema of the lamina propria and intense infiltration of inflammatory cells in the submucosa and muscular layer. The intestinal barrier consists of the intestinal mucus barrier, epithelial barrier and vascular barrier. Among the three layers, the mucus barrier is the first line of defense against harmful organisms, with mucins being the major component of mucus that are secreted mainly by goblet cells. The epithelial barrier is a monolayer cellular barrier tightly bound together by intercellular junctional complexes, including ZOs, occludins, claudins and others (34). We performed AB-NFR staining and immunofluorescence staining to evaluate the expression of mucins and the tight junction protein ZO-1, respectively, and to analyze changes in the intestinal barrier of the ileum. As expected, the expressions of mucins (**Figures 2C–E**) and ZO-1 (**Figures 2F, G**) were significantly decreased in both the ICH and SAP groups compared with the sham-operated control group, indicating the damages of the intestinal mucus and epithelial barrier. Studies have shown that γδT cells in the colon mucosa are the primary producers of early protective interleukin (IL)-17A, which can protect mice from dextran sulfate sodium (DSS)-induced gut injury and leakiness by regulating the cellular localization of the tight junction protein occludin (35, 36). IL-17A has also been reported to increase the expression of claudins in intestinal epithelial cells (37). Thus, both IL-17A and IL-17A-producing γδT cells play important protective roles in maintaining epithelial barriers. Therefore, we also evaluated the expressions of γδ TCRs and IL-17A in ileum tissues. As shown in **Figures 2H-J**, the majority of IL-17A-producing cells were γδ TCRs-expressing small intestinal intraepithelial γδT lymphocytes, since the IL-17A proteins colocalized with γδ TCRs and resided mainly in the paracellular spaces between intestinal epithelial cells. Moreover, the expressions of both γδ TCRs and IL-17A were lower in the ICH and SAP group than those in the sham-operated control group. These data indicate that the SAP rats exhibited the intestinal structure and gut barrier damages and the reductions of the protective intestinal IL-17A-producing γδT cells at 7 days after ICH.

### *Kp* challenge exacerbates gut microbiota dysbiosis in ICH rats

**Figure 3:**
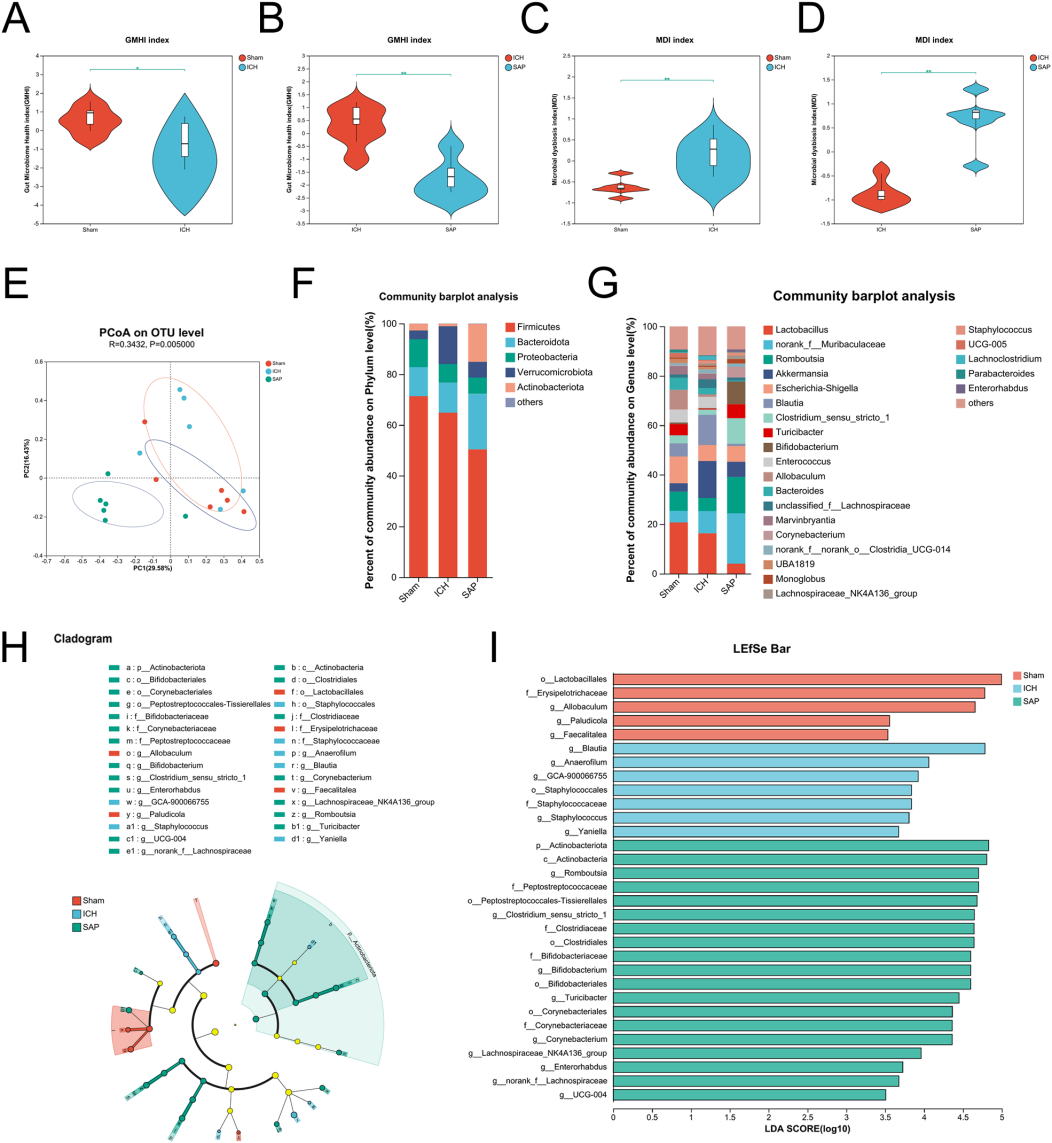
*Kp* challenge exacerbated gut microbiota dysbiosis 7 days after ICH induction. **(A, B)** Comparisons of the GMHI between the ICH and sham-operated control groups **(A)** or between the ICH and SAP groups **(B–D)** Comparisons of the MDI between the ICH and sham-operated control groups **(C)** or between the ICH and SAP groups **(D, E)** PCoA of the microbial communities based on the Bray–Curtis distance at the OTU level. **(F, G)** Relative abundances of bacterial taxa at the phylum **(F)** or genus **(G)** level. Only phyla or genera with an average relative abundance > 1% are shown. **(H)** Cladograms generated by LEfSe indicating differences in the bacterial taxa among the three groups. The color nodes indicate the groups. Red, blue, and green nodes, differential taxa enriched in the sham-operated control, ICH, and SAP groups, respectively. Yellow nodes, taxa with no significant differences among the three groups. **(I)** LEfSe analysis was used to identify the differential bacterial taxa among the three groups. n = 6 rats per group. **(A-D)** Two-tailed Wilcoxon rank-sum test. *P < 0.05 and **P < 0.01. For **(H, I)**, the Kruskal−Wallis H test was used. Only taxa with P values <0.05 and LDA scores greater than 3.5 are shown.

A profound alteration in the gut microbiota composition occurred in *Kp*-challenged mice (38), which was also observed in stroke and SAP patients and corresponding experimental animal models (39–43). Therefore, we performed 16S rRNA sequencing to explore the changes in the gut microbiota in *Kp*-induced SAP rats. The data revealed no significant differences in alpha diversity indices among the sham-operated control, ICH and SAP groups (**Supplementary Figures 2A-F**). We further used the Gut Microbiome Health Index (GMHI) (44) or the Microbial Dysbiosis Index (MDI) (45) to predict the health status or the severity of dysbiosis via OTU-level gut microbiome profiling. The data revealed that the GMHI index of the ICH group was significantly lower than that of the sham-operated control group, while that of the SAP group was further lower (**Figures 3A, B**), indicating that the intestinal health of the SAP rats further deteriorated. In contrast, the MDI index of the ICH group was significantly higher relative to the sham-operated control group, while that of the SAP group was further higher (**Figures 3C, D**), suggesting that the dysbiosis of gut microbiota was further aggravated. Based on the Bray–Curtis distances, principal coordinate analysis (PCoA) also revealed that the gut microbial community structure of the SAP group was clearly separated from those of the sham-operated control and ICH groups (**Figure 3E**). Next, we analyzed alterations in microbial compositions and abundances of specific bacteria at different taxonomic levels. At the phylum level, the relative abundance of *Firmicutes* was significantly decreased in the SAP group compared with the sham-operated control, whereas that of *Actinobacteria* was significantly increased in the SAP group compared to both the sham-operated control and ICH groups. The relative abundance of *Patescibacteria* was significantly different among the three groups and tended to increase in the SAP group but did not differ significantly in pairwise comparisons (**Figure 3F**, **Supplementary Figures 2H-J**). At the genus level, 37 genera differed among the three groups. Among the top 15 differential genera, *Blautia* was decreased in the SAP group compared with the ICH group. *Norank_f:Ruminococcaceae* was decreased in the SAP group compared with sham-operated controls. *Allobaculum* and *Faecalitalea* were depleted in the SAP group. In contrast, *Romboutsia*, *Clostridium_sensu_stricto_1*, *Bifidobacterium*, *Corynebacterium*, and *Enterorhabdus* were increased in the SAP group compared with both the sham-operated control and ICH groups. *Turicibacter* was significantly increased in the SAP group compared to the ICH group. *Lachnospiraceae_NK4A136_group*, *Staphylococcus*, *Roseburia*, *Tyzzerella* and *norank_f:Lachnospiraceae* were significantly different among the three groups and tended to increase in the SAP group but did not differ significantly in pairwise comparisons (**Figure 3G**, **Supplementary Figure 2K**). We further employed linear discriminant analysis (LDA) effect size (LEfSe) analysis to identify the specific bacterial taxa that differed among the three groups. In total, 31 differentially abundant bacterial taxa were identified when the LDA score was >3.5 and are displayed in a cladogram (**Figure 3H**). Among them, *Lactobacillales*, *Erysipelotrichaceae*, *Allobaculum*, *Paludicola*, and *Faecalitalea* were the dominant bacteria in the sham-operated control group; *Blautia*, *Anaerofilum*, *GCA-900066755*, *Staphylococcales*, *Staphylococcaceae*, *Staphylococcus*, and *Yaniella* were the most abundant bacteria in the ICH group; and *Actinobacteriota*, *Actinobacteria*, *Romboutsia*, *Peptostreptococcaceae*, *Peptostreptococcales-Tissierellales*, *Clostridium_sensu_stricto_1*, *Clostridiaceae*, *Clostridiales*, *Bifidobacteriaceae*, *Bifidobacterium*, *Bifidobacteriales*, *Turicibacter*, *Lachnospiraceae_NK4A136_group*, *Corynebacteriales*, *Corynebacteriaceae*, *Corynebacterium*, *Enterorhabdus*, *norank_f:Lachnospiraceae* and *UCG-004* were the dominant bacteria in the SAP group (**Figure 3I**). Taken together, these data indicate that *Kp* challenge exacerbates gut microbiota dysbiosis in ICH rats.

### *Kp* challenge causes profound alterations in the fecal metabolome in ICH rats

**Figure 4:**
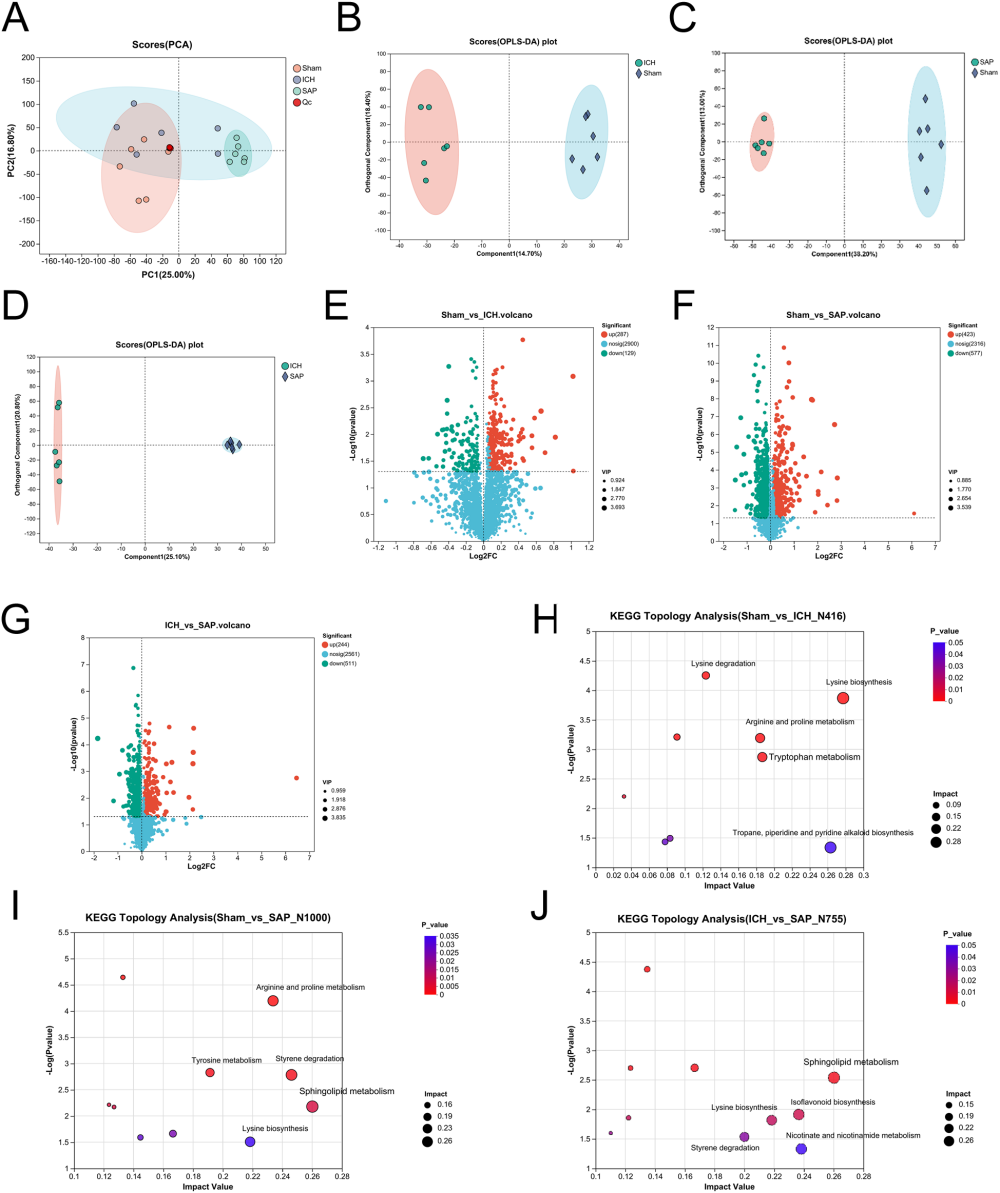
*Kp* challenge caused profound alterations in the fecal metabolic profile 7 days after ICH induction. **(A)** PCA score plot of the overall fecal metabolic profile differences among the sham-operated control, ICH and SAP groups. **(B-D)** OPLS-DA score plots of the sham-operated control vs. ICH **(B)**, sham-operated control vs. SAP **(C)**, and ICH vs. SAP groups **(D)**. The validation plots were obtained from 200 tests. **(E-G)** Volcano plots of the significantly differentially abundant metabolites for the sham-operated control vs. ICH **(E)**, sham-operated control vs. SAP **(F)**, and ICH vs. SAP **(G)** groups screened in positive and negative ion modes. **(H-J)** KEGG topology analyses of the differentially abundant metabolic pathways identified from the comparisons of the sham-operated control vs. ICH **(H)**, sham-operated control vs. SAP **(I)**, and ICH vs. SAP groups **(J)**. Only the top five differentially abundant metabolic pathways are marked according to the impact value. n = 6 rats per group. For **(E-G)**, two-tailed unpaired Student’s t test was used, and P < 0.05 was considered to indicate statistical significance. For **(H-J)**, relative-betweenness centrality was adopted for topology analyses. The Benjamini–Hochberg multiple correction method was used to verify the P values and control the false-positives of the topology results. A corrected P < 0.05 was considered to indicate statistical significance.

*Kp* infection led to a profound change in the cecal metabolic profile (38). Both stroke and SAP also induce metabolic alterations (39, 46, 47). Hence, we adopted an LC−MS/MS-based untargeted metabolomic method to profile changes in the fecal metabolome of SAP rats. We first used unsupervised PCA to evaluate the overall metabolome differences among the three groups and the degree of variability across samples within groups. The data revealed that the metabolic profile of the SAP group was clearly separated from that of the sham-operated control group, whereas the metabolic profile of the ICH group appeared approximately midway between that of the sham-operated control group and the SAP group, and the overall metabolic profile remained more similar to that of the sham-operated control group (**Figure 4A**). We distinguished metabolic differences between groups more precisely and screened the variables that contributed to the separation of each group by employing orthogonal partial least squares discriminant analysis (OPLS-DA). As illustrated in **Figures 4B-D**, the three groups could be clearly separated from each other, indicating that their metabolic profiles were distinct. Differentially abundant metabolites with OPLS-DA VIP>1 and p < 0.05 were subsequently screened between groups and presented in a volcano plot (**Figures 4E–G**). In total, 416 differentially abundant metabolites were identified between the ICH group and the sham-operated control group, with 129 upregulated and 287 downregulated in the ICH group (**Figure 4E**). In addition, 1000 differentially abundant metabolites were found between the SAP group and sham-operated controls, of which 577 were upregulated and 423 were downregulated in SAP rats (**Figure 4F**). Furthermore, compared to those in ICH rats, 755 differentially abundant metabolites were detected in SAP rats, of which 511 were upregulated and 244 were downregulated (**Figure 4G**). We performed KEGG topology analysis to characterize the differentially abundant metabolites in the context of biological pathways and identified tryptophan (Trp) metabolism as one of the top three significantly different metabolic pathways between the sham-operated control and ICH groups (**Figure 4H**). We also identified sphingolipid metabolism as the most significantly different metabolic pathway between the sham-operated control and SAP groups or between the ICH and SAP groups (**Figure 4I, J**), suggesting that ICH dramatically affected Trp metabolism, whereas *Kp* challenge dramatically affected sphingolipid metabolism.

### *Kp* challenge leads to abnormal sphingolipid metabolism and elevated ceramide levels in ICH rats

**Figure 5:**
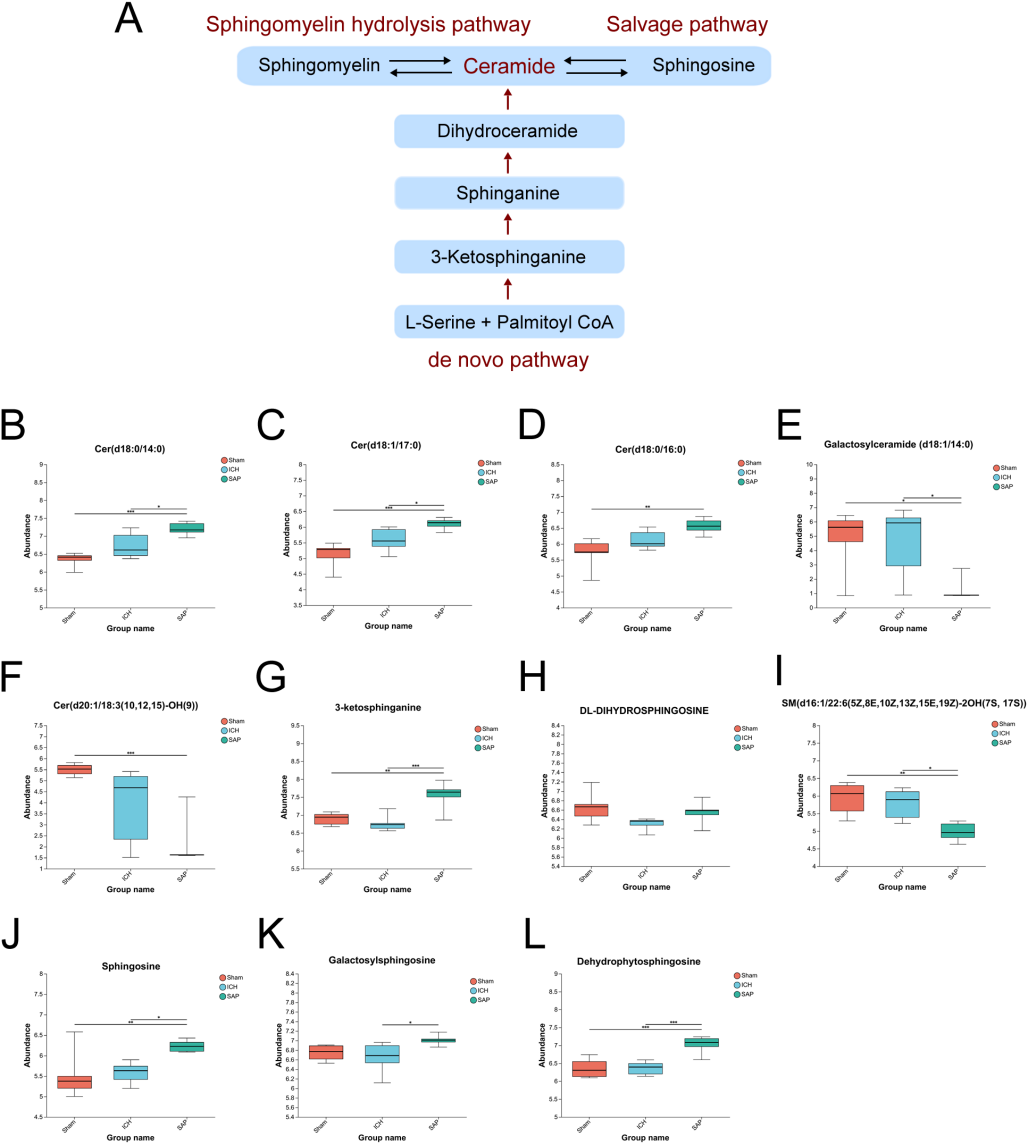
*Kp* challenge led to abnormal sphingolipid metabolism and elevated ceramide levels 7 days after ICH induction. **(A)** Schematic depiction of ceramide biosynthesis via the *de novo*, salvage, and sphingomyelin hydrolysis pathways. **(B-L)** Key differentially abundant metabolites involved in the sphingolipid metabolism pathway among the sham-operated control, ICH and SAP groups. The data are presented as the means ± SDs. n = 6 rats per group. For **(B-L)**, one-way ANOVA and the Tukey−Kramer *post hoc* test were used. *p < 0.05, **p < 0.01, and ***p < 0.001.

Since the analysis of the global fecal metabolic profile revealed that the most prominent altered metabolic pathway in SAP rats was sphingolipid metabolism, we further explored the differentially abundant sphingolipid metabolites. The data revealed that some long-chain ceramides, such as Cer (d18:0/14:0) and Cer (d18:1/17:0), were more abundant in the SAP group than those in the sham-operated control and ICH groups (**Figures 5B, C**), whereas the Cer (d18:0/16:0) level was significantly increased in the SAP group compared with the sham-operated control group (**Figure 5D**). However, that of the glycosylated ceramide derivative galactosylceramide (d18:1/14:0) was significantly decreased in the SAP group compared with both the sham-operated control and ICH groups (**Figure 5E**), whereas that of Cer (d20:1/18:3 (10, 12, 15)-OH (9)) was significantly decreased compared to the sham-operated control group (**Figure 5F**). Ceramides can be synthesized through the *de novo* pathway (**Figure 5A**), which starts from the condensation of L-serine and palmitoyl coenzyme A (CoA) to produce 3-ketosphinganine, which is subsequently reduced to produce sphinganine (dihydrosphingosine). Then, an acyl-CoA chain is bound to form dihydroceramide, which is ultimately converted into ceramide (48). We observed a significantly increased level of 3-ketosphinganine in the SAP group compared to both the sham-operated control and ICH groups (**Figure 5G**); however, although the relative abundance of sphinganine was significantly different among the three groups and tended to be higher in the SAP group than in the ICH group, no significant differences were observed in pairwise comparisons (**Figure 5H**). Ceramides can also be produced through the sphingomyelin (SM) hydrolysis pathway (48). We observed a significantly decreased level of SM(d16:1/22:6(5Z,8E,10Z,13Z,15E,19Z)-2OH (7S, 17S)) in the SAP group than that in the sham-operated control and ICH groups (**Figure 5I**). Ceramides can also be hydrolyzed into sphingosines, which can either be phosphorylated to generate the pleiotropic bioactive metabolite sphingosine-1-phosphate or re-utilized in the salvage pathway to generate ceramides (49). We found that the levels of sphingosine and dehydrophytosphingosine were significantly increased in the SAP group compared with both the sham-operated control and ICH groups (**Figures 5J, L**), whereas that of galactosylsphingosine was more abundant in the SAP group than that in the ICH group (**Figure 5K**). Taken together, these data suggested that *Kp* challenge led to abnormal elevated ceramides levels through the activations of the *de novo*, the sphingomyelinase hydrolysis and the salvage pathways of sphingolipid metabolism.

### *Kp* challenge increases neurotoxic quinolinic acid levels and upregulates the Trp–serotonin–melatonin pathway in ICH rats

**Figure 6:**
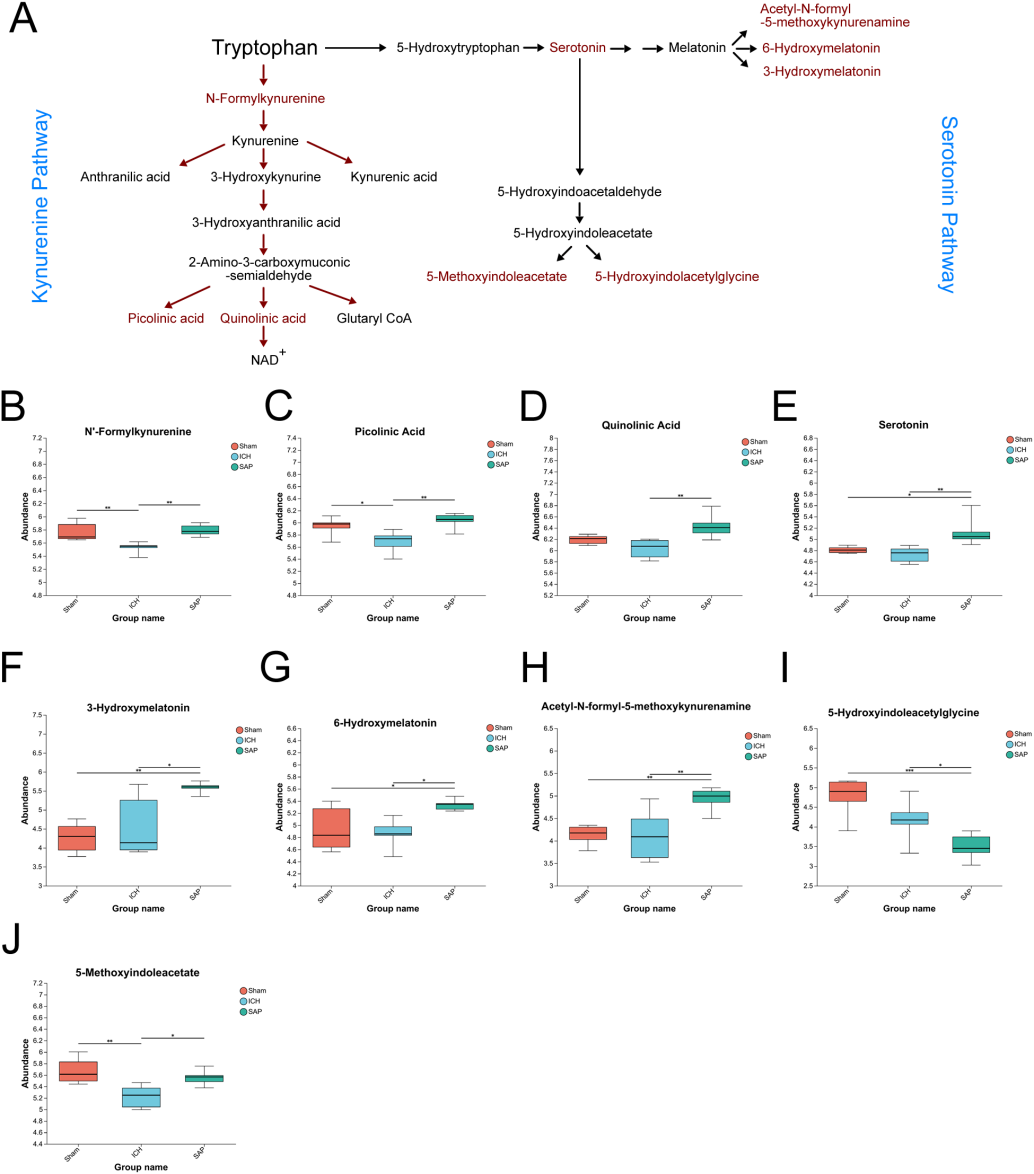
*Kp* challenge caused increased levels of neurotoxic quinolinic acid and an upregulation of tryptophan (Trp)–serotonin–melatonin pathway 7 days after ICH induction. **(A)** Schematic depiction of Trp metabolism via the kynurenine and serotonin pathways. **(B-J)** Key differentially abundant metabolites involved in the Trp metabolism pathway among the sham-operated control, ICH and SAP groups. The data are presented as the means ± SDs. n = 6 rats per group. For **(B-J)**, one-way ANOVA and the Tukey−Kramer *post hoc* test were used. *p < 0.05, **p < 0.01, and ***p < 0.001.

In humans, Trp is an essential amino acid that must be obtained from the diet. In addition to being involved in protein synthesis, Trp can also be degraded through two main pathways (**Figure 6A**): the first is the kynurenine pathway, through which Trp is metabolized into kynurenine, followed by kynurenic acid, anthranilic acid, picolinic acid, QA and other neuroactive intermediates; and the other is the serotonin pathway, through which TRP is metabolized into serotonin and its downstream products, such as melatonin, acetyl-N-formyl-5-methoxykynurenamine, 5-hydroxyindoleacetylglycine and 5-methoxyindoleacetate (50). Since the analysis of the global fecal metabolic profile revealed that ICH dramatically altered Trp metabolism, we further explored the differentially abundant metabolites in this pathway in SAP rats. The data revealed that the levels of the Trp-kynurenine pathway metabolites N’-formylkynurenine and picolinic acid were significantly lower in the ICH group than in the sham-operated control group but were higher in the SAP group (**Figures 6B, C**). However, the level of neurotoxic QA (51) was significantly increased in SAP compared to ICH rats (**Figure 6D**), indicating that ICH led to a significant downregulation of the Trp–kynurenine pathway, whereas *Kp* challenge induced increased neurotoxic QA production in ICH rats. We also found that the levels of the Trp–serotonin–melatonin pathway metabolites serotonin and melatonin derivatives 3-hydroxymelatonin, acetyl-N-formyl-5-methoxykynurenamine and 6-hydroxymelatonin were significantly increased in the SAP group compared with both the sham-operated control and ICH groups (**Figures 6E–H**), whereas the levels of the Trp–serotonin pathway metabolite 5-hydroxyindoleacetylglycine were significantly decreased in the SAP group compared with both the sham-operated control and ICH groups (**Figure 6I**), and the level of 5-methoxyindoleacetate was significantly decreased in the ICH group compared with the sham-operated control group but was increased in the SAP group (**Figure 6J**), indicating that *Kp* challenge induced significant upregulation of the Trp–serotonin pathway characterized by increased levels of melatonin derivatives but decreased 5-hydroxyindoleacetylglycine levels. Taken together, these data suggest that *Kp* challenge increases neurotoxic QA levels and upregulates the Trp–serotonin–melatonin pathway in ICH rats.

### Correlations between the differential genera and differentially abundant sphingolipid or Trp metabolites

**Figure 7:**
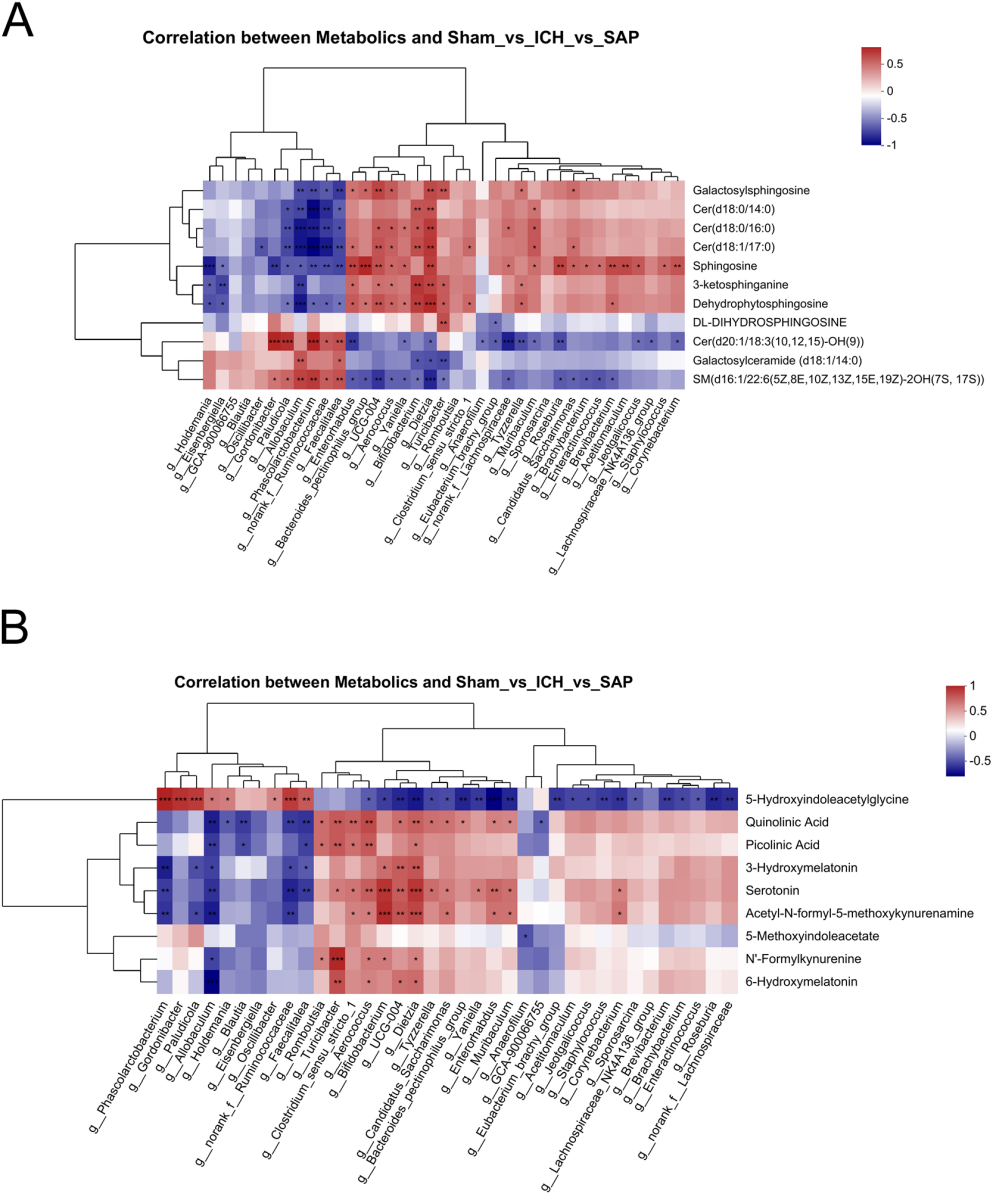
Correlations between differential genera and differentially abundant sphingolipid or Trp metabolites. **(A, B)** Spearman’s correlation analyses between the differential genera and differentially abundant sphingolipid **(A)** or Trp **(B)** metabolites among the sham-operated control, ICH and SAP groups. Each row represents a metabolite, each column represents a genus, and each lattice represents the Spearman correlation coefficient between a genus and a metabolite. Red indicates a positive correlation, whereas blue indicates a negative correlation. Correlations were considered significant when P < 0.05. n = 6 rats per group. *P < 0.05, **P < 0.01, and ***P < 0.001.

We summarized the 37 differential genera among the three groups (P < 0.05, LDA > 2) and performed Spearman’s correlation analyses to further investigate the mechanistic links between gut dysbiosis and the disturbance of sphingolipid metabolism. As shown in **Figure 7A**, some differential genera enriched in *Kp*-induced SAP rats, such as *Dietzia*, *Bifidobacterium*, *Muribaculum*, *UCG-004*, *Aerococcus*, *Enterorhabdus*, *Bacteroides_pectinophilus_group*, *Turicibacter*, and *Clostridium_sensu_stricto_1*, were positively associated with the differentially abundant sphingolipid metabolites upregulated in SAP rats, including Cer(d18:0/14:0), Cer(d18:1/17:0), Cer(d18:0/16:0), 3-ketosphinganine, sphingosine, galactosylsphingosine and dehydrophytosphingosine. However, the differential genera enriched in sham-operated control rats but decreased or even depleted in SAP rats, such as *Allobaculum*, *Faecalitalea*, *norank_f:Ruminococcaceae*, *Gordonibacter*, *Phascolarctobacterium* and *Paludicola*, were negatively associated with the aforementioned differentially abundant metabolites upregulated in SAP rats. Surprisingly, almost all the genera that positively correlated with the sphingolipid metabolites upregulated in SAP rats were negatively correlated with that downregulated in the SAP group, including galactosylceramide (d18:1/14:0), Cer(d20:1/18:3 (10, 12, 15)-OH (9)), SM(d16:1/22:6(5Z,8E,10Z,13Z,15E,19Z)-2OH(7S, 17S)), and vice versa. We also performed Spearman’s correlation analyses to investigate the relationships between the differential genera and the differentially abundant Trp metabolites. As shown in **Figure 7B**, some differential genera enriched in *Kp*-induced SAP rats, such as *Bifidobacterium*, *UCG-004*, *Dietzia*, *Turicibacter*, *Aerococcus*, *Clostridium_sensu_stricto_1*, *Romboutsia*, *Candidatus_Saccharimonas*, *Enterorhabdus*, *Muribaculum*, and *Corynebacterium*, were positively associated with the key differentially abundant Trp metabolites upregulated in SAP rats, including QA, serotonin, acetyl-N-formyl-5-methoxykynurenamine, 3-hydroxymelatonin, and 6-hydroxymelatonin, and other metabolites that were changed in SAP rats, such as N’-formylkynurenine and picolinic acid. However, the differential genera enriched in sham-operated control rats but decreased or even depleted in SAP rats, such as *Allobaculum*, *Faecalitalea*, *norank_f:Ruminococcaceae*, *Gordonibacter*, *Phascolarctobacterium* and *Paludicola*, were negatively associated with the aforementioned differentially abundant Trp metabolites that changed in the SAP group. Notably, almost all the genera that positively correlated with the aforementioned differentially abundant metabolites changed in SAP rats were negatively correlated with 5-hydroxyindoleacetylglycine that downregulated in the SAP group, and vice versa. Taken together, these results suggested that the depletion of *Allobaculum* and *Faecalitalea*, the reduction in *norank_f:Ruminococcaceae*, *Gordonibacter*, *Phascolarctobacterium* and *Paludicola*, and the increase in *Bifidobacterium*, *UCG-004*, *Dietzia*, *Turicibacter*, *Romboutsia*, *Candidatus_Saccharimonas*, *Clostridium_sensu_stricto_1*, *Aerococcus*, *Enterorhabdus*, *Muribaculum, Bacteroides_pectinophilus_group*, *Corynebacterium* and other differential genera in SAP rats are associated with the disordered sphingolipid and Trp metabolism.

### Establishment of a model of ALI complicating ICH via intratracheal inoculation with LPS after ICH

**Figure 8:**
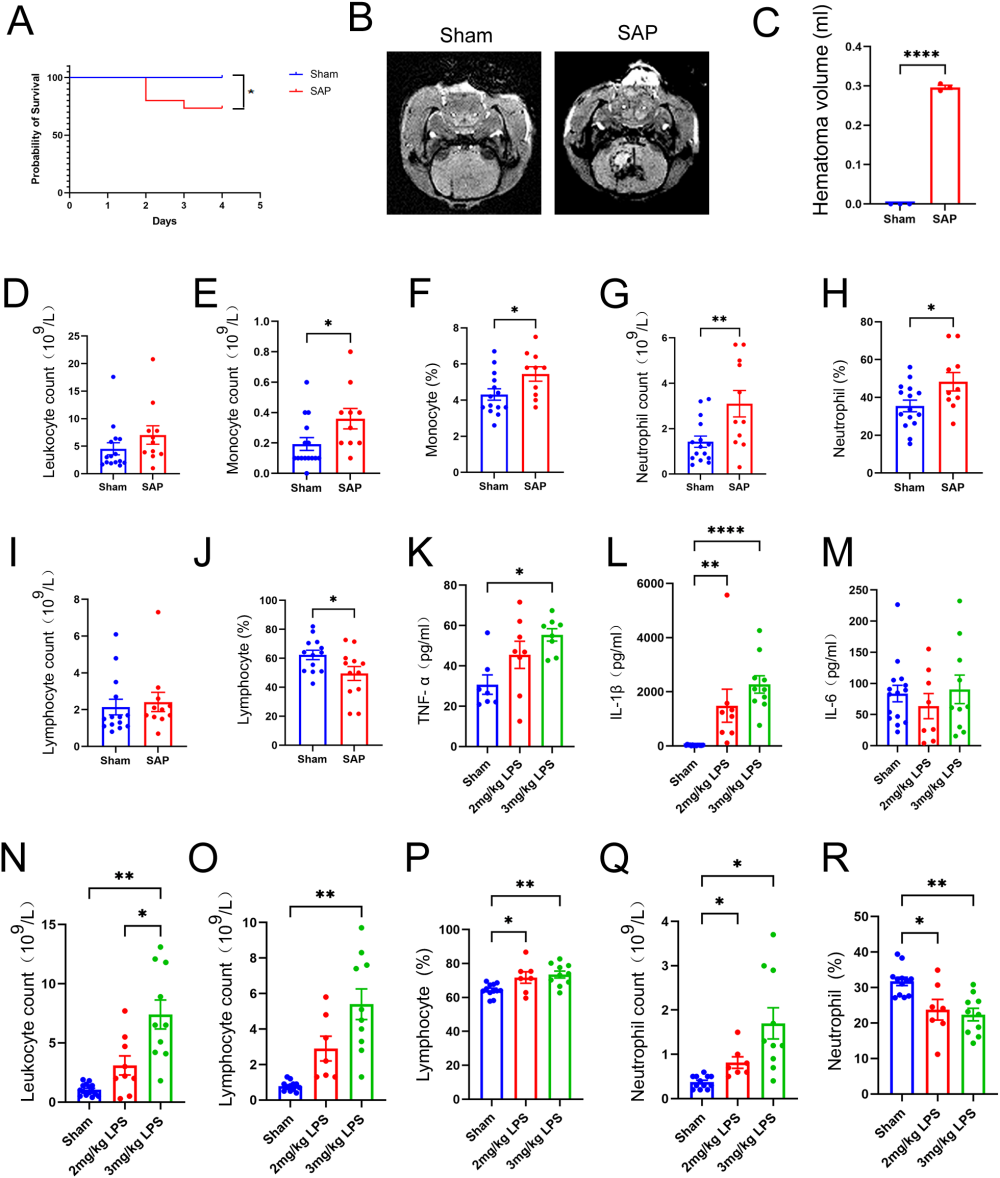
Changes in survival rates, hematoma volume, and peripheral and pulmonary immune responses in rats with LPS-induced ALI complicating ICH at 4 days after ICH induction. **(A)** Survival rates of SAP and sham-operated control rats. Kaplan–Meier analysis with the log-rank test were used. n = 15 rats per group. **(B)** Representative MR images of hematomas in SAP rats. SWI was performed to visualize the hematoma using a 3.0-T MRI scanner. **(C)** Quantification of the hematoma volume in SAP rats, which was calculated using the Coniglobus formula. For **(B, C)**, n = 3 rats per group. **(D-J)** Changes in the number of circulating leukocytes in SAP rats relative to those in sham-operated control rats. **(D)** Total leukocyte count; **(E)** monocyte count; **(F)** proportion of monocytes; **(G)** neutrophil count; **(H)** proportion of neutrophils; **(I)** lymphocyte count; **(J)** proportion of lymphocytes. n = 11–15 rats per group. **(K-M)** ELISAs of TNF-α **(K)**, IL-1β **(L)** and IL-6 **(M)** levels in the serum of sham-operated control, 2 mg/kg LPS-induced or 3 mg/kg LPS−induced SAP rats. n = 7–15 rats per group. **(N-R)** Changes in leukocytes in the BALF of 3 mg/kg LPS−induced SAP rats relative to those in the BALF of 2 mg/kg LPS−induced SAP rats or sham-operated control rats. **(N)** Total leukocyte count; **(O)** lymphocyte count; **(P)** proportion of lymphocytes; **(Q)** neutrophil count; **(R)** proportion of neutrophils. n = 7–15 rats per group. The data are presented as the means ± SEMs. For **(C-J)**, two-tailed unpaired Student’s t test was used; for **(K−M)**, the Kruskal−Wallis H test and Dunnett’s T3 multiple comparison test were used; for **(N, O, Q)**, the Brown−Forsythe test, Welch’s ANOVA and Dunnett’s T3 multiple comparison test were used; for **(P, R)**, one−way ANOVA and Tukey’s multiple comparison test were used. *P<0.05, **P<0.01, and ****P<0.0001. Sham, sham-operated control; SAP, 3 mg/kg LPS−induced ALI complicating ICH model; 2 mg/kg LPS, 2 mg/kg LPS−induced ALI complicating ICH model; 3 mg/kg LPS, 3 mg/kg LPS−induced ALI complicating ICH model.

Due to biosafety concerns, we further established LPS-induced ALI complicating ICH model through intratracheal inoculation of 3 mg/kg LPS after ICH. The survival rate was 73.3% at 4 days after ICH induction (1 day after the LPS inoculation, **Figure 8A**). Significant reductions in body weight were observed on the day of LPS inoculation and at 1 day post-LPS inoculation (at 3 and 4 days after ICH) in the SAP group compared with the sham-operated control group (**Supplementary Figure 3A**). A significant increase in the mNSS was also observed beginning on the day of ICH induction (**Supplementary Figure 3B**). MRI was also performed to detect the lesion locations and measure the volume of hematoma using SWI pulse sequences. The results showed that the injection of 0.5 U type VII collagenase indeed induced an approximately 0.3 ml hematoma volume within the right caudate nucleus at 4 days after ICH (**Figure 8B, C**). Histopathological changes in the brain were also evaluated. As shown in **Supplementary Figure 3C**, SAP rats displayed significant histopathological deterioration, especially moderate infiltration of inflammatory cells in the perihematomal area. Taken together, these data revealed that significant weight loss, serious neurological deficits, and brain damage with perihematomal inflammatory cell infiltration also occurred in LPS-induced ALI complicating ICH rats.

### The peripheral immune response is dysregulated in LPS-induced ALI complicating ICH rats

Immunosuppression after stroke is characterized primarily by a rapid and massive loss of T cells due to apoptosis, which is a key pathological event correlated with increased susceptibility to life-threatening infections such as SAP (9). Moreover, significantly elevated total leukocyte counts, monocyte counts, neutrophil counts and cytokine levels in the blood have been observed in patients with stroke-associated infection compared with those without stroke-associated infection (52). Therefore, we performed a routine blood test to count and classify leukocytes in the blood. The data revealed no significant differences in total leukocyte or lymphocyte counts between the sham-operated control and SAP groups (**Figures 8D, I**). In addition, the numbers and proportions of monocytes and neutrophils were significantly increased in the SAP group compared to the sham-operated control group (**Figures 8E–H**). However, the proportion of lymphocytes was significantly decreased in SAP rats compared to sham-operated control rats, suggesting that immunosuppression also occurred in LPS-induced ALI complicating ICH rats (**Figure 8J**). The upregulation of inflammatory cytokines is an important feature of ALI (53). Therefore, we performed ELISAs to determine the levels of TNF-α, IL-1β and IL-6 in the serum. The data revealed that TNF−α levels were only significantly increased in the 3 mg/kg LPS− but not 2 mg/kg LPS−induced SAP rats compared with the sham-operated control (**Figure 8K**). IL-1β levels were significantly increased in both 2 mg/kg LPS- and 3 mg/kg LPS−induced SAP rats compared with sham-operated control (**Figure 8L**). However, no significant differences in the IL-6 levels were observed among the three groups (**Figure 8M**). Taken together, these data reveal that the peripheral immune response is dysregulated in 3 mg/kg LPS-induced SAP rats, which is characterized by a decreased proportion of lymphocytes, increased numbers and proportions of monocytes and neutrophils, and upregulation of the inflammatory cytokines TNF−α and IL-1β.

### An intense pulmonary inflammatory response and histopathological changes occur in LPS-induced ALI complicating ICH rats

An excessive inflammatory response caused by a quick influx of neutrophils into the lung is a hallmark of ALI and plays a key role in the progression of ALI and ARDS (54). Thus, at 1 day after LPS challenge, the BALF were prepared, and the leukocytes were counted and classified. As shown in **Figure 8N**, the total leukocyte count was significantly increased in 3 mg/kg LPS−induced SAP rats compared with both the sham-operated control and 2 mg/kg LPS−induced SAP rats. The number of lymphocytes was significantly increased only in 3 mg/kg LPS−induced SAP rats (**Figure 8O**). The proportions of lymphocytes and the numbers of neutrophils were significantly increased in both 2 mg/kg LPS- and 3 mg/kg LPS−induced SAP rats (**Figures 8P, Q**), whereas the proportions of neutrophils were significantly decreased in both 2 mg/kg LPS- and 3 mg/kg LPS−induced SAP rats (**Figure 8R**). Pulmonary histopathological changes were also evaluated. As shown in **Supplementary Figure 3D**, 3 mg/kg LPS−induced SAP rats presented marked alveolar wall and bronchiolar wall thickening, alveolar and bronchiolar collapse, peribronchiolar and interstitial edema and intense infiltration of inflammatory cells into the lung interstitium and alveolar spaces, whereas the sham-operated control rats presented no obvious histological alterations. Taken together, these data reveal that an intense pulmonary inflammatory response and histopathological changes occur in LPS-induced ALI complicating ICH rats, and 3 mg/kg LPS is more appropriate than 2 mg/kg LPS for establishing an ALI complicating ICH model.

### Disruption of the intestinal structure and histopathological changes in the ileums in LPS-induced ALI complicating ICH rats

We also performed H&E staining to evaluate the histopathological changes in the ileum sections. As shown in **Supplementary Figure 3E**, at 1 day after LPS challenge, SAP rats presented obvious histological changes characterized by thinner layers of epithelium and muscularis mucosae, shortened villi or even a loss of villi, and intense infiltration of inflammatory cells in the submucosa and muscular layer, whereas sham-operated control rats presented no obvious histological alterations.

## Discussion

In this study, we established a standardized *Kp*-induced Gram-negative bacterial pneumonia complicating ICH model with the following characteristics (1): severe neurological deficits, brain damage, BBB disruption and neuroinflammation in the lesions; (2) lung injury and pneumonia, especially intense infiltration and activation of neutrophils; (3) disruptions of the intestinal structure and gut barrier and downregulations of protective intestinal IL-17A-producing γδT cells; and (4) profound alterations in the fecal microbial community composition and metabolic profile. Due to biosafety concerns, we further established a standardized LPS-induced ALI complicating ICH model that displayed the following characteristics: (1) significant weight loss, severe neurological deficits, brain damage and perihematomal inflammatory cells infiltration; (2) a dysregulated peripheral immune response characterized by a decreased proportion of lymphocytes, increased numbers and proportions of monocytes and neutrophils, and the upregulations of inflammatory cytokines TNF−α and IL-1β; (3) lung injury and a robust inflammatory response in BALF characterized by increased numbers of leukocytes and neutrophils, as well as a decreased number and proportion of leukocytes but a decreased proportion of neutrophils; and (4) disruption of the intestinal structure and histopathological changes in the ileum. Taken together, the changes in peripheral and local immune responses in the blood, lung and ileum; alterations in the fecal microbiota and metabolic profile; and histopathological changes in the brain, lung and gut tissues, all the pathological features mentioned above, supporting the successful establishments of these two models since they are able to duplicate at least some of the mechanisms and consequences of human hemorrhagic stroke and SAP.

SAP is one of the major complications after stroke that worsens functional outcomes (4). However, current prophylactic or therapeutic antibiotic therapies have been proven to be ineffective or need to be used cautiously because of unexpected side effects (7, 8). Thus, more effective therapeutic strategies that target the underlying pathogenesis of SAP are urgently needed. Accumulating evidence suggests that complex multidirectional communications exist among stroke, the gut and lung, which involve the gut microbiota, metabolites, nervous system, neuroendocrine system, immune system, etc (42, 43). The dysbiosis of the gut microbiota is considered closely related to the pathogenesis of lung inflammation (55), lung infection (38, 56), stroke and SAP (11, 39, 40, 43), thus, it is considered a promising therapeutic target (57). In this study, we also observed a profound alteration in the fecal microbiota in rats with *Kp*-induced Gram-negative bacterial pneumonia complicating ICH, which is characterized by the depletion of *Allobaculum*, *Faecalitalea*, the reduction in *norank_f:Ruminococcaceae*, *Gordonibacter*, *Phascolarctobacterium* and *Paludicola*; and the increase in *Bifidobacterium*, *UCG-004*, *Dietzia*, *Turicibacter*, *Romboutsia*, *Candidatus_Saccharimonas*, *Clostridium_sensu_stricto_1*, *Aerococcus*, *Enterorhabdus*, *Muribaculum, Bacteroides_pectinophilus_group*, *Corynebacterium*, etc. Notably, *Allobaculum* in phylum *Firmicutes* is a beneficial bacterium correlated with short-chain fatty acid (SCFA) levels (58) and plays a protective anti-inflammatory role by increasing the levels of SCFAs in the intestine, regulating human metabolism and immunity and protecting intestinal barrier function (59, 60). *Faecalitalea* in phylum *Firmicutes* could also be considered a beneficial bacterium since it can ferment D-glucose, sucrose, D-mannose and raffinose into butyric acid (61), promote postprandial insulin secretion and improve the insulin response in diabetic patients (59). Among the differential genera that significantly enriched in the SAP group, *Turicibacter* is considered a potential pathobiont that positively regulates the associations of unbalanced dietary intake with constipation and total gastrointestinal symptoms in children with autism spectrum disorder (62). *Turicibacter* can also modify host bile acid metabolism (63), the disorder of which could contribute to the pathogenesis of gastrointestinal diseases (64). In addition, *Turicibacter* is also associated with other neurological disorders, such as depression and Parkinson’s disease (65, 66). Both *Dietzia* and *Corynebacterium* belong to the phylum *Actinobacteriota* and are considered opportunistic pathogens (67). *Muribaculaceae* can degrade mucins in the mucus layer and use mucus-derived sugars as crucial nutrients in the gut (68). *Clostridium_sensu_stricto_1* is also regarded as an opportunistic pathogen that causes inflammation, pediatric diarrhea and mucosal injury in the gut (69, 70). Furthermore, some beneficial bacteria, such as *Bifidobacterium*, are enriched in *Kp*-induced SAP rats, which probably reflects the self-regulatory effect to antagonize the gut microbiota dysbiosis, metabolism and immune disorders, as well as other pathological changes, caused by SAP.

In this study, we also observed a profound alteration in the fecal metabolic profile of *Kp*-induced SAP rats, which was characterized mainly by abnormal sphingolipid metabolism, especially elevated ceramide levels; increased neurotoxic QA levels; and upregulation of the Trp–serotonin–melatonin pathway. Sphingolipids are a complex family of compounds that share a common sphingoid base backbone. In addition to serving as vital structural components of the cell membrane, sphingolipids have also been shown to participate in a series of important biological processes, such as cell proliferation, death, adhesion and migration, autophagy, the cell cycle, inflammation, immune responses, angiogenesis, nutrient uptake, metabolism and cellular signaling (71). Dysregulation of sphingolipid metabolism is strongly associated with the development of various diseases, including cancer, diabetes, inflammatory diseases, neurodegenerative diseases, cardiovascular and cerebrovascular diseases (49, 72–75). Among the sphingolipid family members, ceramides are considered central bioactive molecules in sphingolipid metabolism and important second messengers in cellular signal transduction (72). Currently, a growing body of preclinical and clinical evidence supports alterations in sphingolipid profiles, especially ceramide levels, in ischemic stroke patients (72, 76–79). The abnormally elevated ceramide levels seem to be significantly correlated with the risk, clinical severity and poor functional outcomes of ischemic stroke (80, 81). Several studies also support that the S1P signaling pathway plays a critical role in ICH-induced injury and may be a potential therapeutic target (77), and ICH patients have increased serum levels of lactosylceramide and decreased levels of phytosphingosine, suggesting that abnormal sphingolipid metabolism seems to be involved in the pathogenesis of ICH (79); however, alterations in sphingolipid profiles, especially ceramide levels, in subjects with ICH and SAP need to be further elucidated. In this study, we provide the first experimental evidence that the levels of several long-chain ceramides, such as Cer (d18:0/14:0), Cer (d18:1/17:0) and Cer (d18:0/16:0), which are synthesized mainly through abnormal activations of the *de novo*, sphingomyelinase hydrolysis and salvage pathways of sphingolipid metabolism, are upregulated in *Kp*-induced SAP rats, suggesting that abnormal sphingolipid metabolism, especially elevated ceramide levels, may play a critical role in the pathogenesis of SAP.

Trp and its metabolites also participate in regulating a series of important biological processes, such as immunity, metabolism, neurological function and the maintenance of intestinal homeostasis. Aberrant Trp metabolism plays important roles in the pathogenesis of various diseases, including infectious diseases, autoimmune diseases, digestive system diseases, cancers and CNS diseases (50, 82, 83). Currently, many preclinical and clinical studies have implicated Trp metabolism in the occurrence and development of ischemic stroke. However, very few studies have investigated the role of Trp metabolism in the pathogenesis of ICH and SAP (83, 84). In this study, we also provide the first experimental evidence that the neurotoxic QA, the downstream product of the Trp−kynurenine pathway, was significantly upregulated in *Kp*-induced SAP rats compared to the ICH rats. The elevated QA level we detected in the fecal samples from *Kp*-induced SAP rats might reflect an elevated QA level in the brain. QA is produced mainly by the immune activation of microglia and macrophages in the brain. It can also be synthesized by activated peripheral monocytes, which can enter the brain if the brain barriers are disturbed and contribute to excitotoxicity (51). QA acts as an N-methyl-D-aspartate (NMDA) receptor agonist and exerts potent neurotoxic and excitotoxic effects by inducing astrocyte apoptosis and neuronal dysfunction. In addition, QA can act as a gliotoxin, proinflammatory mediator, and prooxidant molecule and can alter the integrity and cohesion of the BBB (85). An abnormally increased QA level may be involved in the pathogenesis of mood disorders and neurodegenerative diseases such as Parkinson’s disease and Alzheimer’s disease (83, 86).

In addition to the kynurenine pathway, Trp can also be degraded through the serotonin pathway, through which Trp is metabolized into serotonin and its downstream products, such as melatonin and 5-methoxyindoleacetate (50). In this study, we found that the levels of serotonin and melatonin metabolites, including 3-hydroxymelatonin, 6-hydroxymelatonin and acetyl-N-formyl-5-methoxykynurenamine, were significantly increased in *Kp*-induced SAP rats, whereas the level of 5-hydroxyindoleacetylglycine was significantly decreased, suggesting that SAP induced a significant upregulation of the Trp–serotonin–melatonin pathway. Among the differentially abundant metabolites mentioned above, serotonin (5-hydroxytryptophan, 5-HT) is a multifunctional signaling molecule that is produced mainly by enterochromaffin cells residing in the intestinal mucosa (87). In addition to serving as the precursor for melatonin synthesis (88), serotonin also plays a pivotal role in regulating gastrointestinal motility, secretion, sensation, intestinal barrier function and inflammation (57). Dysregulation of the serotonin system is associated with various functional gastrointestinal disorders, such as irritable bowel syndrome, Crohn’s disease, and ulcerative colitis (89). Thus, suppressing the biosynthesis of mucosal serotonin seems to be a promising strategy to alleviate inflammation and gastrointestinal disorders. In addition, the differentially abundant metabolites N-acetyl-N-formyl-5-methoxykynuramine, 6-hydroxymelatonin and 3-hydroxymelatonin are produced mainly via the metabolic oxidation of melatonin and are regarded as more stable and effective free radical scavengers (90–92). 6-Hydroxymelatonin is the principal melatonin metabolite and has been shown to be neuroprotective since it can alleviate singlet oxygen- and QA-induced superoxide anion generation and oxidative damage in rat hippocampal homogenates (92). In addition, 6-hydroxymelatonin is able to protect rat liver homogenates from iron-induced lipid peroxidation and tissue injury (93). After ICH, the lysis of erythrocytes increases the iron content and leads to iron overload, which induces toxic hydroxyl radical production and lipid peroxidation and contributes to perihematomal destruction (94). We speculate that the upregulation of melatonin metabolites, especially 6-hydroxymelatonin, may also reflect the self-regulatory mechanism by which *Kp*-induced SAP rats antagonize ICH-induced iron toxicity, lipid peroxidation, and QA-induced neurotoxicity and excitotoxicity.

Stroke and SAP lead to significant alterations in the gut microbiota in both clinical and animal studies, and gut bacteria potentially influence the metabolism and inflammation of the host through the synthesis of various neurotransmitters, neuropeptides, neurotoxic metabolites, SCFAs, indoles and bile acids that enter the systemic blood and travel to local organs to modulate the BBB, mucosal barriers and functions of neurons and immune cells (42, 43, 57). In this study, we observed that the reduction or depletion of some beneficial bacteria, such as *Allobaculum* and *Faecalitalea*, and the increase in several opportunistic pathogens, such as *Turicibacter*, *Dietzia*, *Corynebacterium*, *and Clostridium_sensu_stricto_1*, are associated with disordered sphingolipid and Trp metabolism in *Kp*-induced SAP rats. Previous studies have shown that the gut microbiota can regulate ceramide metabolism (95), and *Lactobacillus* has been reported to be negatively correlated with serum ceramide and sphingomyelin levels in high-fat diet-fed mice (96). Our study provides new experimental evidence that some special bacteria can also regulate sphingolipid metabolism, especially ceramide levels. In addition, some special bacteria and their metabolites have been revealed to mediate the synthesis and metabolism of serotonin in the host, and changes in serotonin levels induced by the gut microbiota can modulate the host immune response and subsequently influence the coping strategy adopted by the host to defend against pathogens or disease (97–100). In turn, intestinal serotonin can promote the colonization of some spore-forming bacteria, such as *Turicibacter*, in the gut, which were previously reported to promote host serotonin biosynthesis (101). Consistent with this study, we also observed the upregulation of both *Turicibacter* and serotonin levels in *Kp*-induced SAP rats (**Figure 7B**); however, the underlying mechanism of action still needs to be further investigated.

Our study has several limitations. First, we only characterized several pathological features of SAP in these two experimental SAP models at a single time point. Dynamic changes need to be explored to better understand the pathogenesis and provide clues for the optimal timing of interventions for SAP patients. Second, we mainly focused on investigating pneumonia, lung injury and systemic inflammation in these two experimental SAP models, whereas the bacterial loads in the lung, BALF and blood, which were previously evaluated in experimental SAP models of both spontaneous pneumonia after ischemic or hemorrhagic stroke and standardized bacterial pneumonia complicating ischemic stroke (9, 10, 19, 21), were not measured in the present study, but will be a future line of investigation in our laboratory. Third, although this study revealed the reduction or depletion of some beneficial bacteria, such as *Allobaculum* and *Faecalitalea*, and the increase in several opportunistic pathogens, such as *Turicibacter*, *Dietzia*, *Corynebacterium*, and *Clostridium_sensu_stricto_1*, in *Kp*-induced SAP rats, which were associated with abnormal sphingolipid metabolism, especially elevated ceramide levels, and disordered Trp metabolism, especially increased neurotoxic QA levels. However, how these differential genera and metabolites participate in regulating a series of important biological processes, such as immunity, neurological function and intestinal homeostasis, which play important roles in the pathogenesis of SAP, still remains to be elucidated. Fourth, we only employed an untargeted metabolomics approach to explore the global fecal metabolic profiles changes and the sphingolipid and Trp metabolism disorders in *Kp*-induced SAP rats, a targeted metabolomics approach will be more appropriate to further verify and exact quantification of the sphingolipid or Trp metabolites. Fifth, the differential genera or the differentially abundant sphingolipid or Trp metabolites we identified in *Kp*-induced SAP rats may not reflect prevailing intestinal flora and metabolite changes in SAP after ICH, additional studies are needed to verify these mechanisms by using Gram-positive bacteria-induced pneumonia, or using clinical SAP samples.

In summary, we present two standardized rat models of post-ICH pneumonia induced by nasal inoculation with 2×10^5^ CFUs of *Kp* or intratracheal inoculation with 3 mg/kg LPS 3 days after ICH. We first characterized several pathological features of these two models to verify that they are able to duplicate at least some of the mechanisms and consequences of human hemorrhagic stroke and SAP. We provide the first experimental evidence that several long-chain ceramides, the central bioactive molecules involved in sphingolipid metabolism; the neurotoxic quinolinic acid, the downstream product of the tryptophan (Trp)-kynurenine pathway; and the Trp–serotonin–melatonin pathway are upregulated in *Kp*-induced SAP rats. We further discovered that the dysregulated gut microbiota is associated with disordered sphingolipid and Trp metabolism. Taken together, these two models may be highly useful for investigating the pathogenesis and screening and optimizing potential treatments for SAP. Moreover, the differential genera and sphingolipid or Trp metabolites identified above seem to be promising therapeutic targets.

## Data Availability

Data supporting the findings of this study are available from the last corresponding author upon reasonable request. The sequencing data have been deposited in the NCBI SRA database under the accession number PRJNA1136706.
