# Supplementary material for: Rat models of postintracerebral hemorrhage pneumonia induced by nasal inoculation with Klebsiella pneumoniae or intratracheal inoculation with LPS
Source: Front Immunol. 2025 Jan 8;15:1477902. doi: 10.3389/fimmu.2024.1477902 (PMC11750689; doi:10.3389/fimmu.2024.1477902)
Supplement: Supplementary file 1 [file DataSheet1.pdf]

# SUPPLEMENTAL MATERIAL TO

## Rat Models of Postintracerebral Hemorrhage Pneumonia Induced by Nasal Inoculation with Klebsiella pneumoniae or Intratracheal Inoculation with LPS

**Running title: Rat Postintracerebral Hemorrhage Pneumonia Models**

Ruihua Wang[1†], Changlian Gan[2†], Rui Mao[3], Yang Chen[4], Ru Yan[5], Geng Li[6*], Tianqin Xiong[7*] and Jianwen Guo[8*]

[1]Research Team of Prevention and Treatment of Cerebral Hemorrhage Applying Chinese Medicine, The Second Affiliated Hospital of Guangzhou University of Chinese Medicine, Guangzhou, China.
[2]School of Traditional Dai Medicine, West Yunnan University of Applied Science, Xishuangbanna, China.
[3]The Second Clinical College of Guangzhou University of Chinese Medicine, Guangzhou, China.
[4]Department of Bioinformatics, State Key Laboratory of Dampness Syndrome of Chinese Medicine, The Second Affiliated Hospital of Guangzhou University of Chinese Medicine, Guangzhou, China.
[5]State Key Laboratory of Quality Research in Chinese Medicine, Institute of Chinese Medical Sciences, University of Macau, Macao, China.
[6]Laboratory Animal Center, Guangzhou University of Chinese Medicine, Guangzhou, China.
[7]School of Pharmaceutical Sciences, Guangzhou University of Chinese Medicine, Guangzhou, China.
[8]State Key Laboratory of Traditional Chinese Medicine Syndrome, Department of Neurology, Guangdong Provincial Academy of Chinese Medical Sciences, Guangdong Provincial Hospital of Chinese Medicine, The Second Affiliated Hospital of Guangzhou University of Chinese Medicine, Guangzhou, China.

[†]These authors contributed equally to this work and share first authorship.

[*] Correspondence:
Geng Li, Ph.D.
E-mail: lg@gzucm.edu.cn
Tianqin Xiong, Ph.D.
E-mail: xiongtq020@gzucm.edu.cn
Jianwen Guo, Ph.D.
E-mail: drguo@gzucm.edu.cn

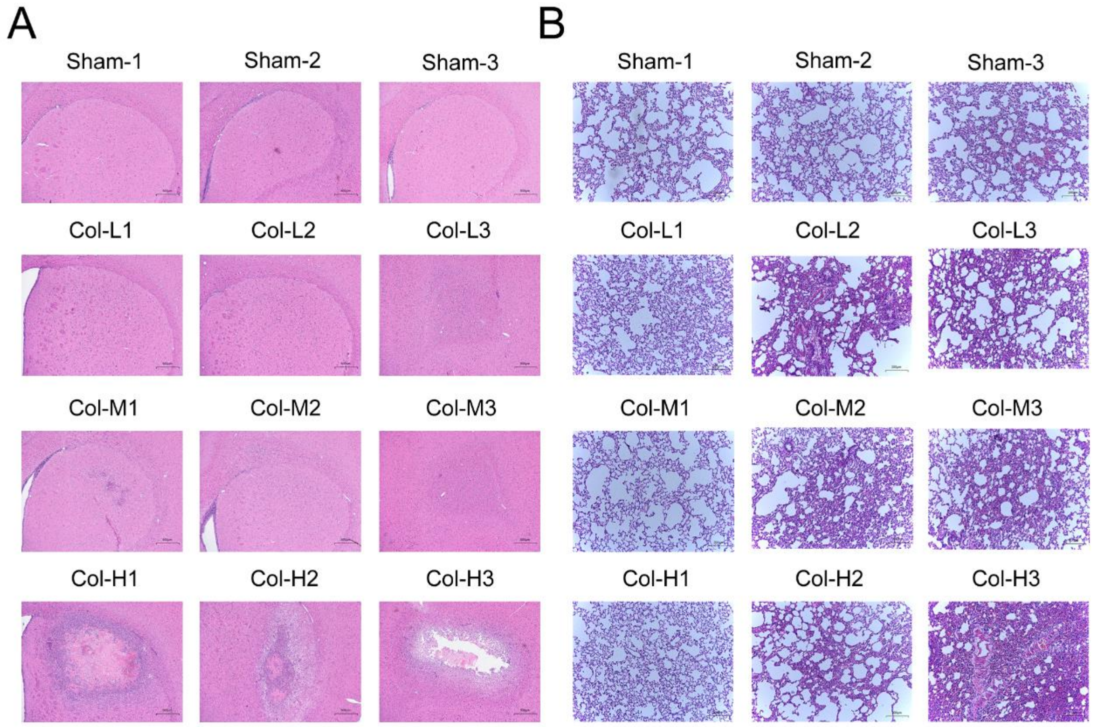


**Supplementary Figure 1. Changes in the histopathology of brain and lung tissues in different doses of type VII collagenase-induced ICH model rats at 7 days after ICH induction.** (**A**, **B**) Representative H&E staining of brain (**A**) and lung (**B**) sections from sham-operated control and different doses of type VII collagenase-induced ICH models. n = 3 rats per group. Scale bars, 500 μm (**A**) and 200 μm (**B**). Sham, sham-operated control; Col-L, M, H, 0.175 U, 0.35 U and 0.7 U type VII collagenase-induced ICH models.

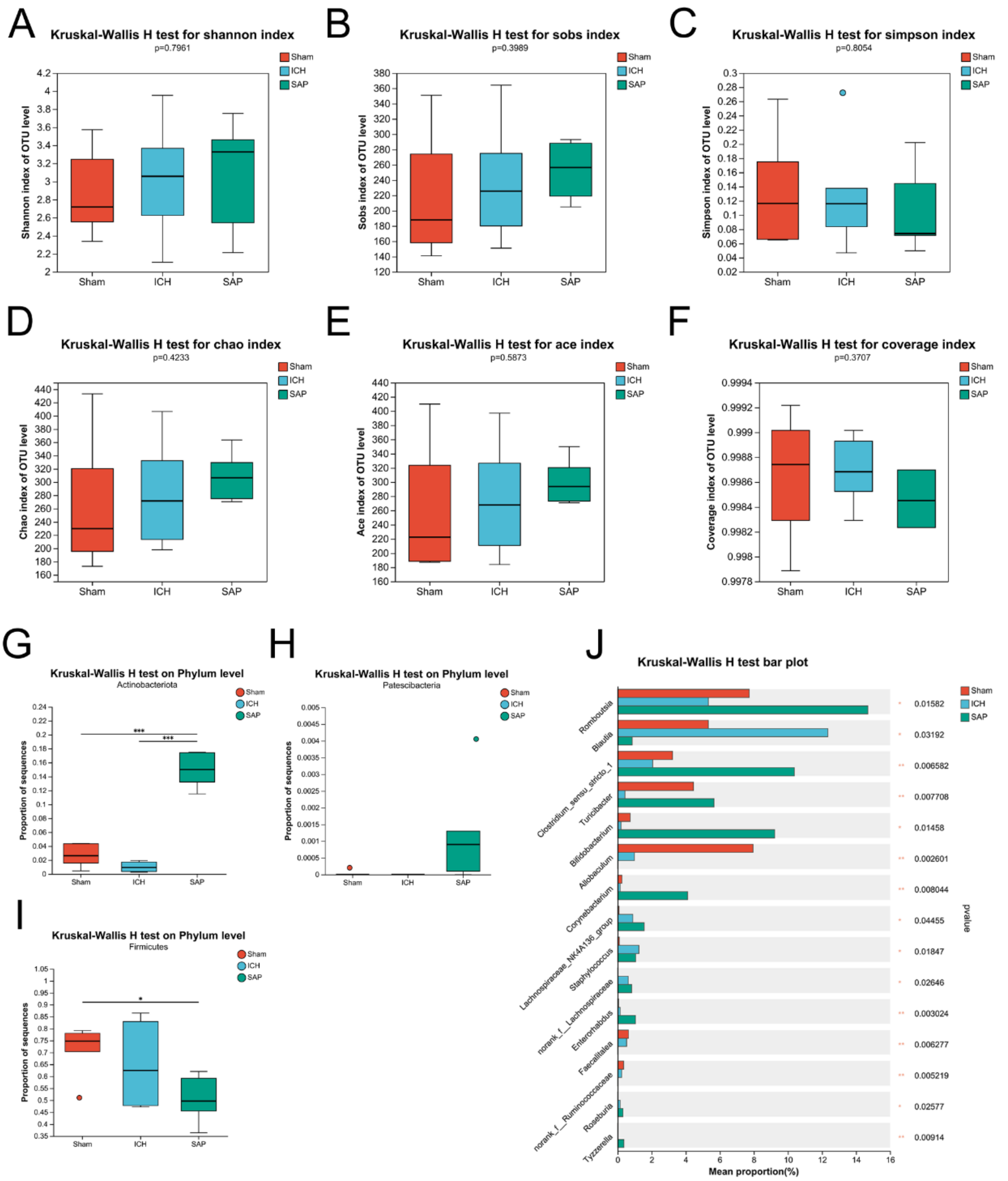


**Supplementary Figure 2. Comparisons of fecal microbial characteristics among the sham-operated control, ICH and SAP rats at 7 days after ICH induction.** (**A** - **F**) Alpha diversity of fecal samples was estimated by shannon (**A**), sobs (**B**), simpson (**C**), chao (**D**), ace (**E**) and coverage (**F**) indices at OTU levels. (**G-I**) The differential bacteria among the three groups at phylum level. (**J**) The differential bacteria among the three groups at genus level. Only the top 15 differential genera are shown. The data are presented as the means ± SDs. n = 6 rats per group. For multiple comparisons, Kruskal-Wallis H test and the Tukey-Kramer post-hoc test were used. *P < 0.05, **P < 0.01, and ***P < 0.001. Sham, sham-operated control; ICH, collagenase-induced intracerebral haemorrhage model; SAP, *Kp*-induced Gram-negative bacterial pneumonia complicating ICH model.

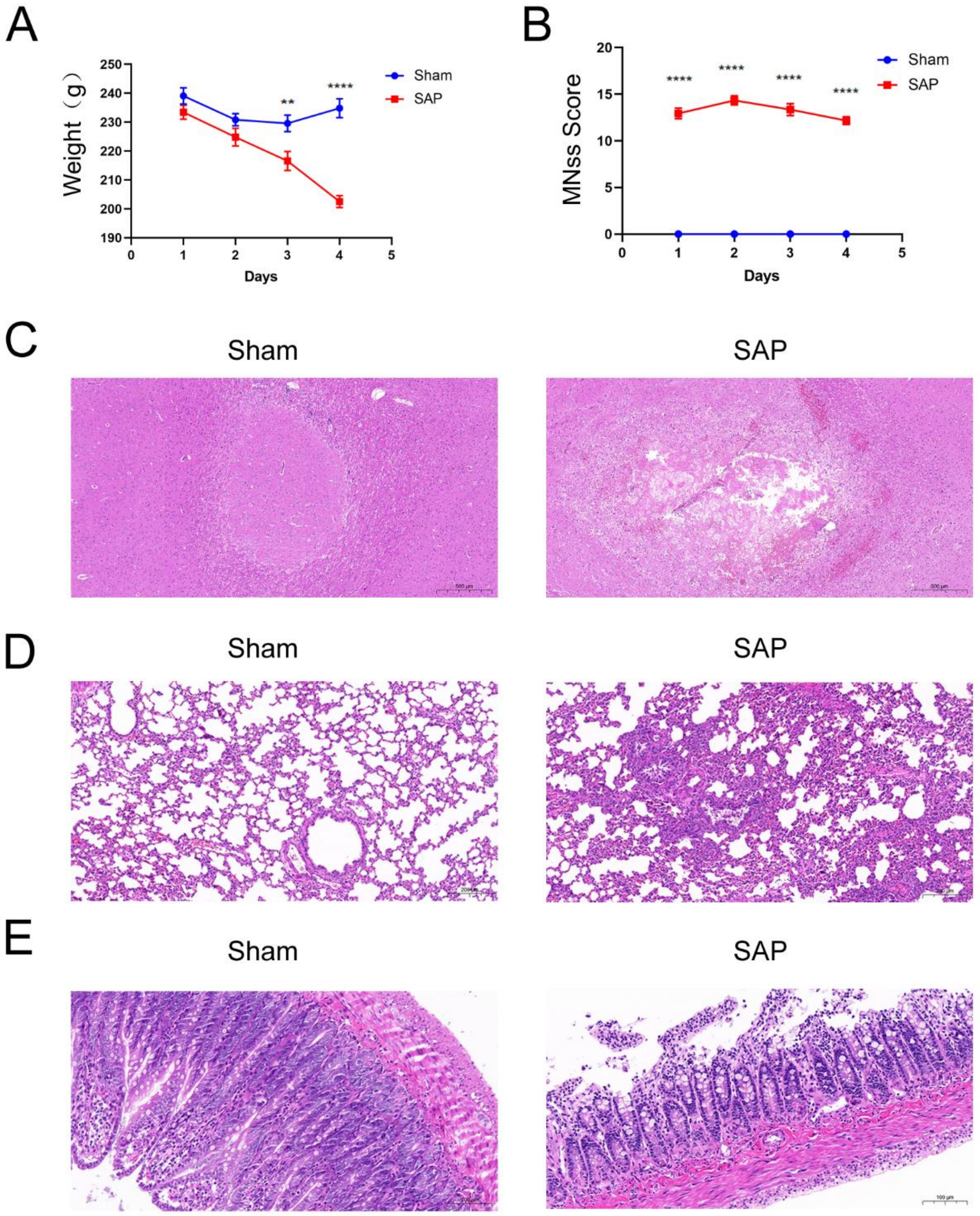


**Supplementary Figure 3. Changes in body weight, mNSS, and the histopathology of brain, lung and ileum tissues in rats with 3 mg/kg LPS-induced ALI complicating ICH at 4 days after ICH induction.** Changes of body weight (**A**) and mNSS (**B**) in SAP and sham-operated control groups. n = 11-15 rats per group. (**C-E**) Representative images of H&E staining of brain (**C**), lung (**D**) and ileum (**E**) sections from SAP and sham-operated control rats at 4 days after ICH induction (1 day after LPS challenge). n = 4-6 rats per group. Scale bars, 500 µm (**C**), 200 µm (**D**) and 100 µm (**E**). The data are presented as the means ± SEMs. For (**A**, **B**), two-tailed unpaired Student's t test was used. **P<0.01 and ****P<0.0001 vs. sham-operated control. Sham, sham-operated control; SAP, 3 mg/kg LPS-induced ALI complicating ICH model.
